# Supplementary material for: Combining genome-wide association studies highlight novel loci involved in human facial variation
Source: Nat Commun. 2022 Dec 20;13:7832. doi: 10.1038/s41467-022-35328-9 (PMC9767941; doi:10.1038/s41467-022-35328-9)
Supplement: Supplementary file 1 — Supplementary Information [file 41467_2022_35328_MOESM1_ESM.pdf]

Supplementary Information for "Combining Genome-wide Association Studies Highlights Novel Loci Involved in Human Facial Variation"

Ziyi Xiong[1, 2, #], Xingjian Gao[3, 4, #], Yan Chen[1, 3, #], Zhanying Feng[5], Siyu Pan[3], Haojie Lu[2, 6], Andre G. Uitterlinden[2, 6], Tamar Nijsten[7], Arfan Ikram[2], Fernando Rivadeneira[2, 6, 8], Mohsen Ghanbari[2], Yong Wang[5], Manfred Kayser[1, *], Fan Liu[1, 3, *]

1. Department of Genetic Identification, Erasmus MC, University Medical Center Rotterdam, Rotterdam, the Netherlands.
2. Department of Epidemiology, Erasmus MC, University Medical Center Rotterdam, Rotterdam, the Netherlands.
3. CAS Key Laboratory of Genomic and Precision Medicine, Beijing Institute of Genomics, Chinese Academy of Sciences, Beijing, China.
4. National Clinical Research Center of Kidney Diseases, Jinling Hospital, Nanjing, Jiangsu, China.
5. CEMS, NCMIS, HCMS, MDIS, Academy of Mathematics and Systems Science, Chinese Academy of Sciences, Beijing, China.
6. Department of Internal Medicine, Erasmus MC, University Medical Center Rotterdam, Rotterdam, the Netherlands.
7. Department of Dermatology, Erasmus MC, University Medical Center Rotterdam, Rotterdam, the Netherlands.

8. Department of Oral and Maxillofacial Surgery, Erasmus MC, University Medical Center Rotterdam, Rotterdam, the Netherlands.

#. These authors contributed equally.

*. These authors jointly supervised this work.

*. Correspondence: Department of Genetic Identification, Erasmus MC, University Medical Center Rotterdam, Rotterdam, the Netherlands; Email: Manfred Kayser: m.kayser@erasmusmc.nl, Fan Liu: f.liu@erasmusmc.nl.

Supplementary Note 1

Other 12 novel loci with supportive evidences were described below. Their facial traits association maps is available in Supplementary Fig. 15.

An intronic variant (rs57839456) of *ZNF718* at 4p16.3 was study-wide significant in C-GWAS ($p=3.3 \times 10^{-8}$), although not replicated ($p>0.05$). The G allele of rs57839456 is mainly associated with an upward lifted mouth. The CNCC network analysis found that the REs in the vicinity of face-associated SNPs at this locus regulate several nearby genes including *ZNF732*, another member of the zinc figure protein family. Face-associated SNPs at 4p16.3 also strongly colocalized with eQTLs of *ZNF718* in Muscle_Skeletal (PP4=0.97) and colocalized with nearby genes in multiple other tissues (PP4>0.9). The members of zinc figure protein family function as transcription factors that can regulate a broad variety of developmental and cellular processes.

At 2q32.1, the lead SNP rs10773002, located 162kbp upstream of *ZC3H15*, was nominally significantly replicated (p=0.017). The A allele of rs57839456 is associated with multiple facial phenotypes and resulting a wider and shorter face. Face-associated SNPs in this locus colocalized with eQTLs of *RP11–410E4.1* in multiple tissues (PP4>0.8). As a member of zinc finger protein family and widely expressed in bone marrow and brain, *ZC3H15* function as a transcription factors that can regulate a broad variety of developmental and cellular processes, which may be involved in facial morphogenesis.

At 1q42.3, the lead SNP rs2055127 is located in the intron of *TBCE*. The C allele

of lead SNP rs2055127, minor in Europeans (*f*=0.49) and Asians (*f*=0.37) but major in Africans (*f*=0.65), was associated with multiple facial phenotypes and resulting a reduced facial width and an increased facial length. The REs in the vicinity of the face-associated SNPs at this locus regulate several nearby genes including *TBCE* in the CNCC sub-network. Face-associated SNPs in this locus strongly colocalized with eQTLs of *TBCE* in Pituitary and Adipose_Subcutaneous (PP4>0.95). SNPs in *TBCE* have been associated with body height[1], type 2 diabetes[2] and mathematical ability[3] in previous GWAS.

At 11q12.2, the lead SNP rs174582 is located in the intron of *FADS2*, and was nominally significantly replicated (p=0.033). The G allele of rs174582 is mainly associated with a lower nasion position. The CNCC network analysis found that the REs in the vicinity of face-associated SNPs at this locus regulate *FADS2* and several nearby genes. Face-associated SNPs in this locus strongly colocalized with eQTLs of *FADS2* and nearby genes in Pituitary (PP4=0.98) and other tissues. The protein encoded by *FADS2* is the desaturase enzymes, a member of the fatty acid desaturase (FADS) gene family, which plays a role in lipid metabolism. SNPs in *FADS2* have been associated with high-density lipoprotein cholesterol levels in previous GWAS[4].

An intronic variant (rs2812241) of *DLEU7* at 13q14.3 was significantly replicated (p=0.000014). The T allele of rs2812241 is mainly associated with an increased nose width. Face-associated SNPs in this locus strongly colocalized with eQTLs of *DLEU7–AS1* in multiple brain-relevant tissues and Adipose (PP4>0.9). The

production of *DLEU7–AS1* is the antisense RNA 1 of *DLEU7*. The latter one is an established gene relevant to body height and hip circumference, as the SNPs in *DLEU7* have been associated with these traits in a large number of previous GWAS.

At 6q27, the lead SNP rs34485638 is located in the intron of *TBP*. The T allele of rs34485638 is associated with multiple facial phenotypes and resulting a longer face. The REs in the vicinity of the face-associated SNPs at this locus regulate *TBP* in the CNCC sub-network. The protein encoded by *TBP* is involved in many biological processes relevant to regulatory, e.g., transcription by RNA polymerase II

At 1p36.12, the lead SNP rs11462489 is located in the intron of *EIF4G3*. The A allele of rs11462489 is associated with multiple facial phenotypes and resulting a shorter nose an increased eyes width. The CNCC network analysis found that the REs in the vicinity of face-associated SNPs at this locus regulate several nearby genes. The protein encoded by *EIF4G3* is thought to be part of eukaryotic translation initiation factor 4F complex, which plays a role in regulatory through enables translation initiation factor activity.

At 2q34, the lead SNP rs7557972, located 100kbp downstream of *PTH2R*, was nominally significantly replicated (p=0.044). The T allele of rs7557972 is associated with multiple facial phenotypes and resulting a shorter nose an increased distances between eyes. Face-associated SNPs in this locus colocalized with eQTLs of a nearby gene *PIKFYVE* in skin-relevant tissues. The protein encoded by *PIKFYVE* is an enzyme, which involved in regulating cytoskeletal functions, membrane trafficking and receptor signaling.

For 4q34.3, 15q14, 15q21.2, and 15q22.2, we found no functional supportive evidence from CNCC network analysis and eQTL colocalization analysis. However, four lead SNPs in these regions were both nominally significantly replicated (p=0.047~0.02). The lead SNP rs17278459 at 4q34.3 is located in the intron of *TENM3*. The G allele of rs17278459 is mainly associated with a narrower and longer face. SNPs in *TENM3* have been associated with appearance and nerve relevant traits, i.e., adolescent idiopathic scoliosis[5], educational attainment[6] and neuroticism[7]. The protein encoded by *TENM3* is involved in the regulation of neuronal development. The lead SNP rs2937979 at 15q14 is located 7.5kbp upstream of *TMCO5A*. The G allele of rs2937979 is mainly associated with a smaller mouth and an increased distance between eyes and nose. SNPs in *TMCO5A* have been associated with appearance relevant traits, i.e., body height[1]. The lead SNP rs8025172 at 15q21.2 is located in the intron of *ATP8B4*. The C allele of rs8025172 is mainly associated with a smaller nose and a reduced distance between nose and mouth. SNPs in *ATP8B4* have been associated with appearance relevant traits, i.e., skin pigmentation[8] and aging[9], in previous GWAS. The lead SNP rs8030235 at 15q22.2 is located 301kbp downstream of *RORA*. The T allele of rs8030235 is mainly associated with a larger mouth and less protrusion in nose. SNPs in *RORA* have been associated with appearance relevant traits, i.e., body height[1] and male-pattern baldness[10], in previous GWAS. The protein encoded by *RORA* has been shown to interact with *NM23-2*, a nucleoside diphosphate kinase involved in organogenesis and differentiation.

Supplementary Method A

We derived $\mathbf{w^T}$ when the off-diagonal elements of $\boldsymbol{\Psi}$ is not 0 using Lagrange multiplier λ in minimizing $\mathrm{Var}(\beta_c)$ subject to the constraint $\mathbf{w^T b} = 1$, i.e., we construct the Lagrangian function:

$$\mathcal{L}(\mathbf{w}, \lambda) = \mathrm{Var}(\beta_c) - \lambda(\mathbf{w^T b} - 1) = \mathbf{w^T C w} - \lambda(\mathbf{w^T b} - 1)$$

For w_i, $i \in \{1,2,\dots,K\}$, let $\nabla_{w_i,\lambda}\mathcal{L}(\mathbf{w}, \lambda) = 0$. Therefore,

$$\frac{\partial \mathcal{L}}{\partial w_i} = 2(\mathbf{w^T C_i} - w_i C_{ii}) + 2 w_i C_{ii} - \lambda = 0$$

Then we have $2\mathbf{w^T C}_i = \lambda$, i.e., $2\mathbf{w^T C} = \lambda \mathbf{b^T}$, $\mathbf{w^T}$ can be derived as $\mathbf{w^T} = \lambda \mathbf{b^T C^{-1}}/2$.

With the constraint $\mathbf{w^T b} = 1$, we have

$$\mathbf{w^T} = \frac{\mathbf{b^T C^{-1}}}{\mathbf{b^T C^{-1} b}} \qquad (5)$$

Equation (5) is equivalent to Equation (4) when the $\boldsymbol{\Psi}$ is an identity matrix,

$$\mathbf{w^T} = \frac{\mathbf{b^T C^{-1}}}{\mathbf{b^T C^{-1} b}} = \frac{\mathbf{b^T V^{-1}}}{\mathbf{b^T V^{-1} b}} = \frac{\boldsymbol{\nu} \circ \boldsymbol{\nu}}{\mathbf{b^T}(\boldsymbol{\nu} \circ \boldsymbol{\nu})^\mathbf{T}} \text{ or } \mathbf{w^T} = \frac{\mathbf{n}}{\mathbf{b^T n^T}}$$

Thus, the IVW (4) is a special case of the revised weight in (5).

Supplementary Method B

We have $\mathbf{G_{all}} = \text{EbICoW}(\mathbf{G}_i, \mathbf{G}_j, \mathbf{\Psi}, \mathbf{\Pi_{all}}, \mathbf{h_{all}})$, $\mathbf{G_{sig}} = \text{EbICoW}(\mathbf{G}_i, \mathbf{G}_j, \mathbf{\Psi}, \mathbf{\Pi_{sig}}, \mathbf{h_{sig}})$ and $\mathbf{G_{stb}} = \text{EbICoW}(\mathbf{G}_i, \mathbf{G}_j, \mathbf{\Psi}, \mathbf{\Pi_{stb}}, \mathbf{h_{stb}})$. The *optimize* selects the optimal EbICoW results from three configurations of π and $\mathbf{h}$. Consider L as a cut-off to filter SNPs with significant effect and function *count* as counting the number of SNPs satisfying the conditions. Typically, $5/M < L < 50/M$, default of L is set to 3×10^{-6}. The *optimize* is defined as below:

$n_{all} = \text{count}(\mathbf{P_{all}} < L)$; $n_{sig} = \text{count}(\mathbf{P_{sig}} < L)$; $n_{stb} = \text{count}(\mathbf{P_{stb}} < L)$;

$n_{all-uni} = \text{count}((\mathbf{P}_i < L)|(\mathbf{P}_j < L)\&(\mathbf{P_{all}} < L))$;

$n_{all-inter} = \text{count}((\mathbf{P}_i < L)\&(\boldsymbol{P}_j < L)\&(\mathbf{P_{all}} < L))$;

$n_{sig-uni} = \text{count}((\mathbf{P_i} < L)|(\mathbf{P}_j < L)\&(\mathbf{P_{sig}} < L))$;

$n_{sig-inter} = \text{count}((\mathbf{P}_i < L)\&(\mathbf{P}_j < L)\&(\boldsymbol{P}_{sig} < L))$;

$n_{stb-uni} = \text{count}((\mathbf{P}_i < L)|(\mathbf{P}_j < L)\&(\mathbf{P_{stb}} < L))$;

$n_{stb-inter} = \text{count}((\mathbf{P}_i < L)\&(\mathbf{P}_j < L)\&(\mathbf{P_{stb}} < L))$;

if $(n_{all} \geq n_{sig})\&(n_{all-uni} \geq n_{sig-uni})\&(n_{all-inter} \geq n_{sig-inter})$ {

    if $(n_{all} \geq n_{stb})\&(n_{all-uni} \geq n_{stb-uni})\&(n_{all-inter} \geq n_{stb-inter})$ {Return $\mathbf{G_{all}}$};

    else {Return $\mathbf{G_{stb}}$}};

else if $(n_{sig} \geq n_{stb})\&(n_{sig-uni} \geq n_{stb-uni})\&(n_{sig-inter} \geq n_{stb-inter})$ {Return $\mathbf{G_{sig}}$};

else {Return $\mathbf{G_{stb}}$};

Supplementary Method C

The *evaluate* make decision whether to accept combined result from *optimize* via evaluating the power of $\mathbf{G_E}$ by counting the number of SNP with significant effect (take L as cut-off) and robustness of $\mathbf{G_E}$ by estimating the equivalent sample size (*Ess*, can be estimated by $\mathrm{E}(1/(2f(1-f)se^2))$ using genome-wide SNPs, where f is allele frequency and se is standard error of a SNP. Consider O as an arbitrary parameter of user choice requesting that the number of significant signals from EbICoW is at least O times of that from the Wald test and that from the adjusted MinGWAS. Typically, $O \geq 1$, default of O is set to 1.1. Another request is that the *Ess* of EbICoW after combination should be larger than the weighted sum of *Ess* of the two GWAS before combination. For this, we set the weight of the GWAS with larger *Ess* to 1 and arbitrarily set the weight of the GWAS with smaller *Ess* to R. The R parameter is of user choice. Typically, $0 \leq R \leq 1$, default of R is set to 0.1. The *evaluate* is defined as below:

$n_E = \mathrm{count}(\mathbf{P_E} < L)$; $n_W = \mathrm{count}(\mathbf{P_W} < L)$;

$n_A = \mathrm{count}(\mathrm{Tippett}(\mathbf{P_A}, \mathrm{getNtest}(\mathbf{P_A})) < L)$; # see Supplementary Method D

if $(n_E \geq O * \mathrm{Max}(n_W, n_A))\&(Ess_E \geq \mathrm{Max}(Ess_i + R * Ess_j, R * Ess_i + Ess_j))$ {

Return TRUE};

else {Return FALSE};

Supplementary Method D

The core functions for distribution calibration involve $\text{getNtest}(\mathbf{P})$, $\text{Tippett}(\mathbf{P}, NT)$, and $\text{getCoef}(\mathbf{P})$. The $\text{getNtest}(\mathbf{P})$ and $\text{Tippett}(\mathbf{P}, NT)$ are used for correcting all intermediate results to make them comparable with each other at the same α. The $\text{getNtest}(\mathbf{P})$ is to estimate the number of independent tests NT in a vector of simulated p-values $\mathbf{P}$ of length NS, as $NT = \alpha_{\text{nominal}}/\text{quantile}(\mathbf{P}, \alpha_{\text{nominal}})$. Each element of $\mathbf{P}$ is simulated from a series of dependent tests under the null, e.g., from each simulation in generating $\mathbf{SP}$. Then Tippett's method is applied so that $\mathbf{P}_{\mathbf{adj}} = 1 - (1 - \mathbf{P})^{NT}$. The observed p-values satisfying $\mathbf{P}_{\mathbf{adj}} \leq \alpha_{\text{nominal}}$ are considered as nominally significant after multiple testing correction for all GWASs.

The $\text{getCoef}(\mathbf{P})$ is used to calibrate an unknown distribution to a known distribution to ensure that all final p-values from C-GWAS follow the uniform distribution under the null. The idea is to adjust through a quantile-specific coefficient function (**Qcoef**) reflecting the relation between the uniform distribution and any given unknown distribution. In other words, we take the simulated non-uniform null as the input and the uniform distribution as the target and obtain the correspondence between the input and the target for all quantiles using locally estimated scatterplot smoothing (LOESS)[11]. LOESS is a very flexible method for modeling complex processes for which no theoretical models exist. More specifically, for a given non-uniform null $\mathbf{P}$ that are generated by NS simulations, the **Qcoef** between the input and the target is obtained using the *getCoef* for all quantiles, as $\text{Qcoef} = \text{LOESS}(\frac{\text{rank}(\mathbf{P})}{NS} \circ \mathbf{RP} \sim \frac{\text{rank}(\mathbf{P})}{NS})$, where $\mathbf{RP}$ is

constructed using the reciprocal of each elements of $\text{sort}(\mathbf{P})$, i.e., $\mathbf{RP} = (1/\text{sort}(\mathbf{P})_1, 1/\text{sort}(\mathbf{P})_2, \dots, 1/\text{sort}(\mathbf{P})_{NS})$, and *sort* order $\mathbf{P}$ as the same order used in *rank*. Therefore, **Qcoef** is a LOESS fitted model that can predict quantile specific coefficients for any given non-uniform null. Then the observed p-value vector from M SNPs in application $\mathbf{OP}$ is adjusted by $\mathbf{OP_{adj}} = \mathbf{OP} \circ \text{predict}(\text{rank}(\mathbf{OP})/M, \mathbf{Qcoef})$.

For all intermediate results from i-EbICoW, we can calculate a genome-wide significant threshold in final C-GWAS p-values as $\alpha \times \text{getNtest}(\mathbf{SP}_{\boldsymbol{Q+1}}) \times \text{predict}(\alpha_{\text{EbICoW}}, \text{getCoef}(\mathbf{minSP}))$, where $\alpha_{\text{EbICoW}} = \text{count}(\mathbf{minSP} < (\alpha \times \text{getNtest}(\mathbf{SP}_{\boldsymbol{Q+1}})))/NS$. For the genome-wide significant SNP in at least one i-EbICoW combination, two adjusted factor represent the multiple testing and calibration burden from *getNtest* and *getCoef* based-correction respectively. The relaxed threshold is data specific and can be considered as the suggestive study-wide significance threshold of C-GWAS.

Supplementary Method E

We can use a series ($n = A$) of the quantiles $\boldsymbol{\eta}$, e.g., $(\frac{1}{A+1}, \frac{2}{A+1}, \ldots, \frac{A}{A+1})$ of $\mathbf{T} \circ \mathbf{T}$ as $\boldsymbol{\gamma}$ to estimate I, where $\boldsymbol{\gamma} = (\gamma_1, \gamma_2 \ldots, \gamma_A)$ and $\gamma_a = \text{quantile}(\mathbf{T} \circ \mathbf{T}, \eta_a)$. Ideally, $\boldsymbol{\eta}$ can be expressed by I, A, p and $\boldsymbol{\gamma}$ as below,

$$\boldsymbol{\eta} = \mathrm{F}_{\chi_1^2}\left(\frac{\boldsymbol{\gamma}}{1+I}\right) \times (1-p) + \mathrm{F}_{\chi_1^2}\left(\frac{\boldsymbol{\gamma}}{1+I+A}\right) \times p$$

Because both A and p can be expressed by functions of I and $\mathbf{T}$ using the moments of $\mathbf{T}$ as below, $\boldsymbol{\eta}$ can be expressed only by I, $\mathbf{T}$ and $\boldsymbol{\gamma}$.

$$p = 1/\left(\frac{\mathrm{Var}(T_m^2) - 2(\mathrm{E}(T_m^2))^2}{3(\mathrm{E}(T_m^2) - 1 - I)^2} + 1\right)$$

$$A = \frac{\mathrm{E}(T_m^2) - 1 - I}{p} = \left(\frac{\mathrm{Var}(T_m^2) - 2(\mathrm{E}(T_m^2))^2}{3(\mathrm{E}(T_m^2) - 1 - I)} + \mathrm{E}(T_m^2) - 1 - I\right)$$

Thus I can be determined when the deviation between derived $\boldsymbol{\eta}$ and expected $\boldsymbol{\eta}$ is minimal, where both A and p can be expressed by I and $\mathbf{T}$ as above,

$$I = \underset{I \in [1, \mathrm{E}(T_m^2)]}{\arg\min} \left(\sum_{a=1}^{A} \left(\mathrm{F}_{\chi_1^2}\left(\frac{\gamma_a}{1+I}\right) \times (1-p) + \mathrm{F}_{\chi_1^2}\left(\frac{\gamma_a}{1+I+A}\right) p - \eta_a\right)^2\right)$$

The minimization is effectively achieved by applying a half-interval search algorithm to the searching space of I from 1 to $\mathrm{E}(T_m^2)$. Consider the searching space $S \in [1, \mathrm{E}(T_m^2)]$, let $\varepsilon = 10^{-4}$ and the function $\text{getdev}(x)$ be calculating the deviation between derived $\boldsymbol{\eta}$ and expected $\boldsymbol{\eta}$ when I is set as x. The process of the *getI* is described as below,

$\mathbf{s} = (1, \mathrm{E}(T_m^2))$; while $\mathrm{Max}(\mathbf{s}) - \mathrm{Min}(\mathbf{s}) > \varepsilon$ {

$\mathbf{s} = \text{quantile}(\mathbf{s}, (0, 0.25, 0.5, 0.75, 1))$;

$id = \text{which}(\mathrm{Min}(\text{getdev}(s_1), \text{getdev}(s_2), \text{getdev}(s_3), \text{getdev}(s_4), \text{getdev}(s_5)))$;

$\mathbf{s} = (s_{\mathrm{Max}(id-1,1)}, s_{\mathrm{Min}(id+1,5)})$}; $s = (\mathrm{Max}(\mathbf{s}) + \mathrm{Min}(\mathbf{s}))/2$;

The inflation factor is estimated as s.

Supplementary Method F

Scenario 1: EbICoW is originated from the traditional inversed variance weighting based meta-analysis with three extensions. The first extension (5) is to introduce $\mathbf{\Psi}$ to obtain a stable null when the background correlation deviates from 0. The second extension (7) is to introduce the effect vector $\mathbf{h}$ to improve power. To compare the power of (5) and (7), we set $N = 50{,}000$, $K = 2$, $p = 0.1$, $\pi \in (-1, 1)$, and the grid was set as $\psi \in \{-0.2, 0, 0.5\}$, $se_1^2/se_2^2 \in \{1{,}4\}$, $\mathrm{E}(\chi^2) \in \{1.1, 1.3, 1.6\}$, note pairs of $\mathrm{E}(\chi^2)$ results in 6 pairs of GWAS.

Scenario 2: The third extension (8) is to adaptively adjust the combination direction according to the relationship of π and ψ by changing the sign directions of the elements in the effect covariance matrix (5). To compare the power of (7) and (8), we set $N = 50{,}000$, $K = 2$, $p = 0.1$, $\pi \in (-1, 1)$, and the grid was set as $\psi \in \{-0.2, 0, 0.5\}$, $se_1^2/se_2^2 \in \{1{,}4\}$, $\mathrm{E}(\chi^2) \in \{1.1, 1.3, 1.6\}$, note pairs of $\mathrm{E}(\chi^2)$ results in 6 pairs of GWAS.

Scenario 3: The key idea of C-GWAS is a combined use of the EbICoW (8) and the Wald (9) to provide the optimal power. To compare power of EbICoW and Wald, we set $N = 50{,}000$, $K = 2$, $p = 0.1$, and the grid was set as ψ and $\pi \in \{-0.98, -0.9, -0.8, \ldots, -0.1, 0, 0.1, \ldots, 0.8, 0.9, 0.98\}$, $\mathrm{E}(\chi^2) \in \{(1.3, 1.3), (1.2, 1.6)\}$.

Scenario 4: For each SNP, TWT proposes the best subset of phenotypes by applying the Wald test to all subsets determined under a series of preset thresholds. The Wald combines all GWASs using the Wald test, thus representing the largest subset of the complete set. The MinGWAS takes the subset with the minimal p-value,

representing the smallest subset of the complete set. To compare power of TWT, Wald and MinGWAS, we set $N = 50{,}000$, $K = 10$, $p = 0.1$, $\pi \in (0,1)$, $\mathbf{r} = (0.5, 0.2, 0.1, 0.05, 0.02, 0.01, 0.005, 0.002, 0.001)$, and the grid was set as $\psi \in \{0, 0.5\}$, $\mathrm{E}(\chi^2) \in \{\text{pattern } 1 \text{ to } 4\}$. For pattern 1, one GWAS with true effect $\mathrm{E}(\chi^2) = 2$ and nine GWASs under the null $\mathrm{E}(\chi^2) = 1$ were simulated; for pattern 2, three GWASs with true effect $\mathrm{E}(\chi^2) \in \{1.6, 1.3, 1.1\}$ and seven GWASs under the null were simulated; for pattern 3, six GWASs with true effect $\mathrm{E}(\chi^2) \in \{1.3, 1.2, 1.2, 1.1, 1.1, 1.1\}$ and four GWASs under the null were simulated; for pattern 4, all ten GWASs were simulated with true effect $\mathrm{E}(\chi^2) = 1.1$. p-values from TWT and the MinGWAS of ten GWAS were adjusted using *getNtest* and *Tippett* function.

Scenario 5: to compare power of EbICoW, TWT, MTAG and MinGWAS, in two GWAS scenario we set $N = 50{,}000$, $K = 2$, $p = 0.1$, $\pi \in (-1,1)$, and the grid was set as $\psi \in \{-0.2, 0, 0.5\}$, $se_1^2/se_2^2 \in \{1,4\}$, $\mathrm{E}(\chi^2) \in \{1.1, 1.3, 1.6\}$, note pairs of $\mathrm{E}(\chi^2)$ results in 6 pairs of GWAS. In multiple GWAS scenario we set $N = 50{,}000$, $K = 10$, $p = 0.1$, $\pi \in [0,1]$, $\mathbf{r} = (0.5, 0.2, 0.1, 0.05, 0.02, 0.01, 0.005, 0.002, 0.001)$, and the grid was set as $\psi \in \{0,0.5\}$, $\mathrm{E}(\chi^2) \in \{\text{pattern } 1 \text{ to } 4\}$. Pattern configuration is the same as used in Scenario 4. p-values from TWT, MTAG and the MinGWAS were adjusted using *getNtest* and *Tippett* function.

Scenario 6: To test inflation control, we set $N = 1{,}000{,}000$, and add an additional independent inflation to GWAS with mean χ^2 value $I \in [0, 0.3]$. The simulated parameter grid was set as, proportion of SNP with true effect, $prop_{\mathrm{TE}} \in \{0.001, 0.01, 0.1\}$; mean χ^2 of GWAS with true effect, $\mathrm{E}(\chi^2_{\mathrm{TE}}) \in \{1.1, 1.3, 1.6\}$. Genomic

control lambda value is calculated as median χ^2 of GWAS divided by one-degree freedom χ^2 at p-value of 0.5.

Scenario 7: To test estimation of ψ, we set $N = 1{,}000{,}000$, $K = 2$, $\psi \in (-1, 1)$, $\pi \in (-1, 1)$, and the grid was set as, proportion of SNP with true effect, $prop_{\mathrm{TE}} \in \{0.001, 0.01, 0.1\}$; mean χ^2 of GWAS with true effect, $\mathrm{E}(\chi^2) \in \{1, 1.1, 1.3, 1.6\}$, note pairs of $\mathrm{E}(\chi^2)$ results in 9 pairs of GWAS. Other two compared correlation are calculated by: 1) Pearson correlation coefficient between two GWAS using all test statistics. 2) Pearson correlation coefficient between two GWAS using the SNPs which are nominally non-significant (p>0.05) in both GWASs

Scenario 8: The α of EbICoW and TWT is affected only by two factors, i.e., the estimation errors of inflation s and the estimation errors of the background correlation ψ. To test α of all intermediate results from EbICoW and TWT, we set $N = 10{,}000$, $\mathbf{r} = (0.5, 0.2, 0.1, 0.05, 0.02, 0.01, 0.005, 0.002, 0.001)$, $N \in \{2,3, \dots ,100\}$, $\psi \in \{0,0.5\}$, and three levels of random noise added to s and ψ with variance 10^{-4}, 6×10^{-5} and 2×10^{-5}. Note that we added the same noise to s and ψ to avoid unnecessary computations. For each of the 1,188 cells in the parameter grid, 1000 replicates were carried out. For each analysis of combining GWASs, a false positive result is recorded if that analysis produces at least one SNP with $p < 0.05/10000$. The α is calculated as the count of false positive results divided by 1,000. p-values from TWT were adjusted using *getNtest* and *Tippett* function.

Scenario 9: The last step of C-GWAS is to adjust all p-values using the *getCoef* function. This is to ensure that the final results from C-GWAS under the null

follows the uniform distribution. To examine whether or not the C-GWAS results under the null follows the uniform distribution, we simulated summary statistics of 30 GWAS simulated under the null, each consisting of 10,000 SNPs without any true effect. We evaluated the null distribution of C-GWAS and MinGWAS under two different $\psi \in \{0, 0.5\}$. All pairs of GWASs were simulated to have the same ψ.

Supplementary Reference

1. Kichaev, G. *et al.* Leveraging Polygenic Functional Enrichment to Improve GWAS Power. *Am J Hum Genet* **104**, 65-75 (2019).
2. Vujkovic, M. *et al.* Discovery of 318 new risk loci for type 2 diabetes and related vascular outcomes among 1.4 million participants in a multi-ancestry meta-analysis. *Nat Genet* **52**, 680-691 (2020).
3. Lee, J.J. *et al.* Gene discovery and polygenic prediction from a genome-wide association study of educational attainment in 1.1 million individuals. *Nat Genet* **50**, 1112-1121 (2018).
4. Hoffmann, T.J. *et al.* A large electronic-health-record-based genome-wide study of serum lipids. *Nat Genet* **50**, 401-413 (2018).
5. Liu, J. *et al.* The coexistence of copy number variations (CNVs) and single nucleotide polymorphisms (SNPs) at a locus can result in distorted calculations of the significance in associating SNPs to disease. *Hum Genet* **137**, 553-567 (2018).
6. Okbay, A. *et al.* Polygenic prediction of educational attainment within and between families from genome-wide association analyses in 3 million individuals. *Nat Genet* **54**, 437-449 (2022).
7. Luciano, M. *et al.* Association analysis in over 329,000 individuals identifies 116 independent variants influencing neuroticism. *Nat Genet* **50**, 6-11 (2018).
8. Hernandez-Pacheco, N. *et al.* Identification of a novel locus associated with skin colour in African-admixed populations. *Sci Rep* **7**, 44548 (2017).
9. McCartney, D.L. *et al.* Genome-wide association studies identify 137 genetic loci for DNA methylation biomarkers of aging. *Genome Biol* **22**, 194 (2021).
10. Yap, C.X. *et al.* Dissection of genetic variation and evidence for pleiotropy in male pattern baldness. *Nat Commun* **9**, 5407 (2018).
11. Cleveland, W.S. Robust Locally Weighted Regression and Smoothing Scatterplots. *Journal of the American Statistical Association* **74**, 829-836 (1979).

Supplementary Figures

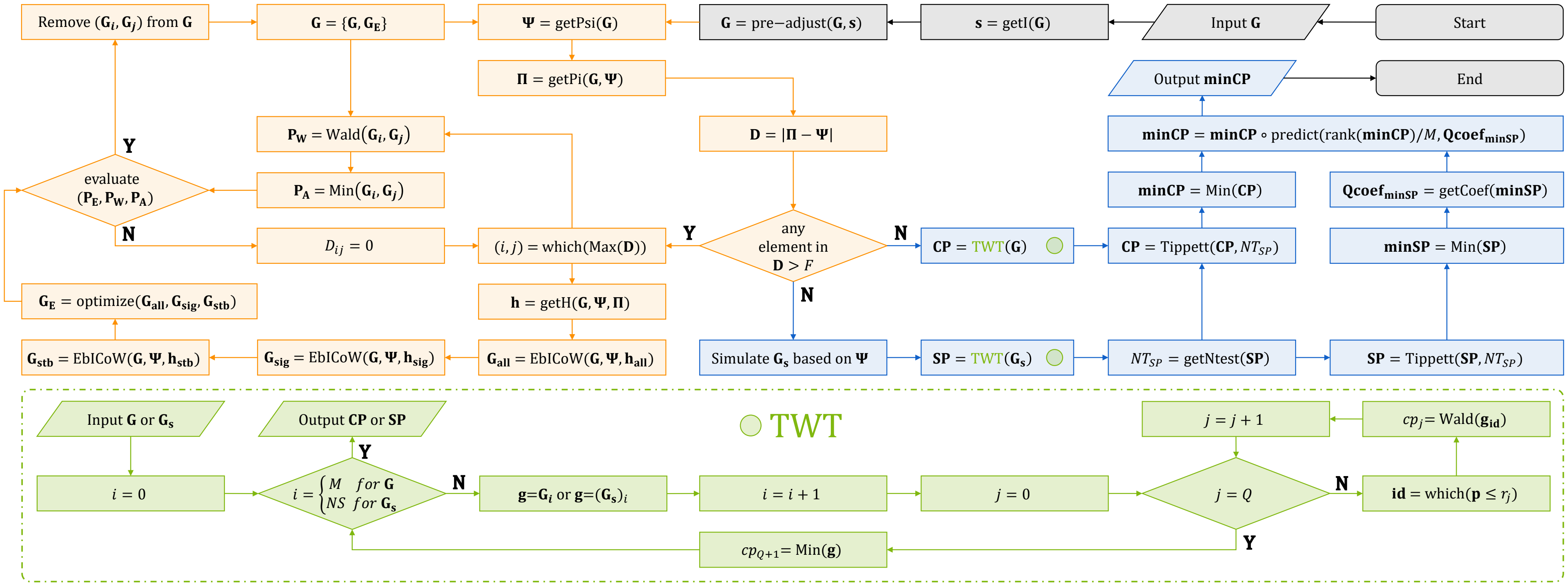

Supplementary Figure 1. C-GWAS workflow.

I-EbICow module is highlighted with orange color. TWT and distribution calibration module is highlighted with blue color and TWT is detailed illustrated in a separate flowchart with green color. Details regarding to the functions involved in the flowchart are described in the method section.

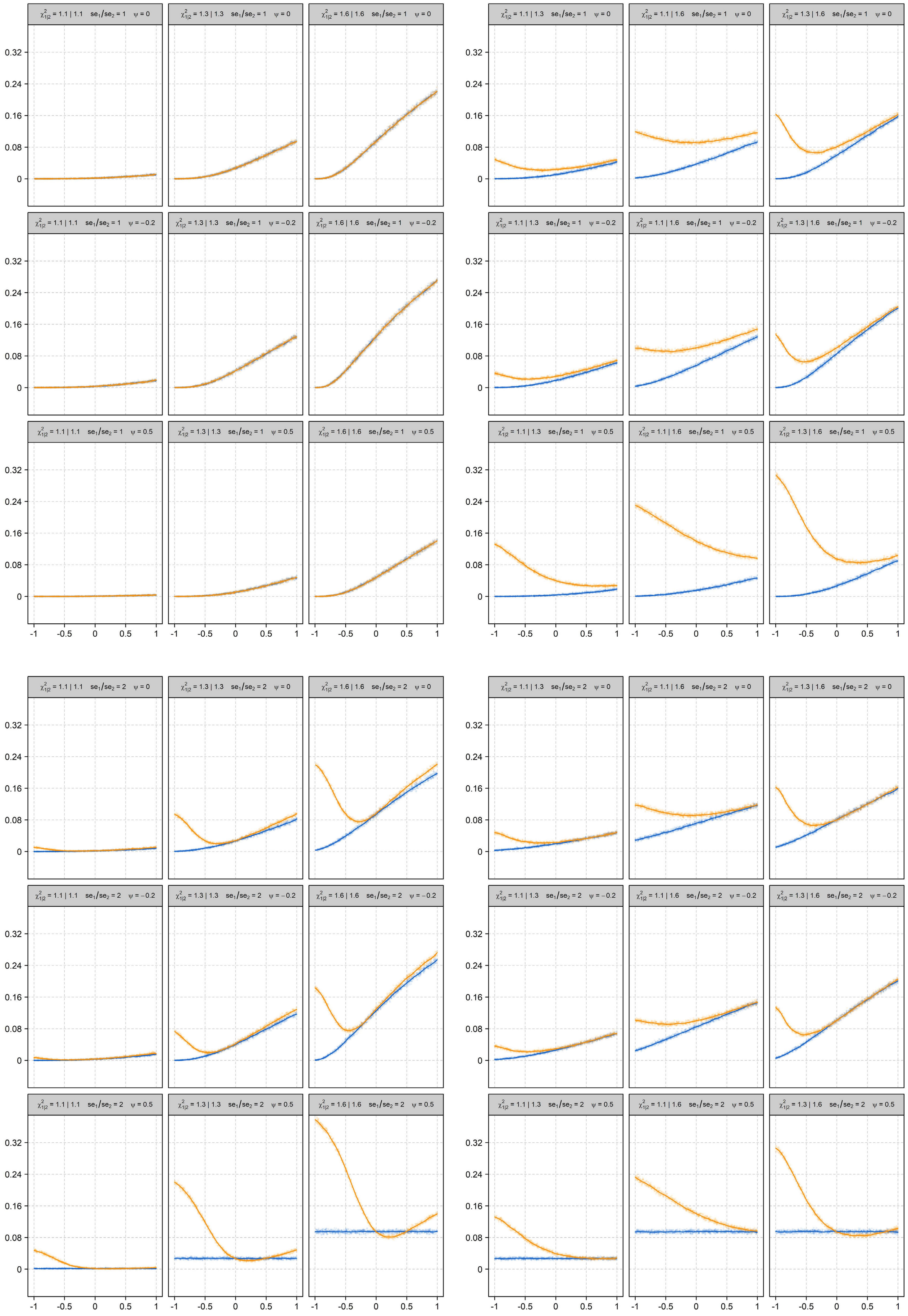
$\chi^2_{1|2} = 1.1 \mid 1.1$ $se_1/se_2 = 1$ $\psi = 0$
$\chi^2_{1|2} = 1.3 \mid 1.3$ $se_1/se_2 = 1$ $\psi = 0$
$\chi^2_{1|2} = 1.6 \mid 1.6$ $se_1/se_2 = 1$ $\psi = 0$
$\chi^2_{1|2} = 1.1 \mid 1.3$ $se_1/se_2 = 1$ $\psi = 0$
$\chi^2_{1|2} = 1.1 \mid 1.6$ $se_1/se_2 = 1$ $\psi = 0$
$\chi^2_{1|2} = 1.3 \mid 1.6$ $se_1/se_2 = 1$ $\psi = 0$
$\chi^2_{1|2} = 1.1 \mid 1.1$ $se_1/se_2 = 1$ $\psi = -0.2$
$\chi^2_{1|2} = 1.3 \mid 1.3$ $se_1/se_2 = 1$ $\psi = -0.2$
$\chi^2_{1|2} = 1.6 \mid 1.6$ $se_1/se_2 = 1$ $\psi = -0.2$
$\chi^2_{1|2} = 1.1 \mid 1.3$ $se_1/se_2 = 1$ $\psi = -0.2$
$\chi^2_{1|2} = 1.1 \mid 1.6$ $se_1/se_2 = 1$ $\psi = -0.2$
$\chi^2_{1|2} = 1.3 \mid 1.6$ $se_1/se_2 = 1$ $\psi = -0.2$
$\chi^2_{1|2} = 1.1 \mid 1.1$ $se_1/se_2 = 1$ $\psi = 0.5$
$\chi^2_{1|2} = 1.3 \mid 1.3$ $se_1/se_2 = 1$ $\psi = 0.5$
$\chi^2_{1|2} = 1.6 \mid 1.6$ $se_1/se_2 = 1$ $\psi = 0.5$
$\chi^2_{1|2} = 1.1 \mid 1.3$ $se_1/se_2 = 1$ $\psi = 0.5$
$\chi^2_{1|2} = 1.1 \mid 1.6$ $se_1/se_2 = 1$ $\psi = 0.5$
$\chi^2_{1|2} = 1.3 \mid 1.6$ $se_1/se_2 = 1$ $\psi = 0.5$
$\chi^2_{1|2} = 1.1 \mid 1.1$ $se_1/se_2 = 2$ $\psi = 0$
$\chi^2_{1|2} = 1.3 \mid 1.3$ $se_1/se_2 = 2$ $\psi = 0$
$\chi^2_{1|2} = 1.6 \mid 1.6$ $se_1/se_2 = 2$ $\psi = 0$
$\chi^2_{1|2} = 1.1 \mid 1.3$ $se_1/se_2 = 2$ $\psi = 0$
$\chi^2_{1|2} = 1.1 \mid 1.6$ $se_1/se_2 = 2$ $\psi = 0$
$\chi^2_{1|2} = 1.3 \mid 1.6$ $se_1/se_2 = 2$ $\psi = 0$
$\chi^2_{1|2} = 1.1 \mid 1.1$ $se_1/se_2 = 2$ $\psi = -0.2$
$\chi^2_{1|2} = 1.3 \mid 1.3$ $se_1/se_2 = 2$ $\psi = -0.2$
$\chi^2_{1|2} = 1.6 \mid 1.6$ $se_1/se_2 = 2$ $\psi = -0.2$
$\chi^2_{1|2} = 1.1 \mid 1.3$ $se_1/se_2 = 2$ $\psi = -0.2$
$\chi^2_{1|2} = 1.1 \mid 1.6$ $se_1/se_2 = 2$ $\psi = -0.2$
$\chi^2_{1|2} = 1.3 \mid 1.6$ $se_1/se_2 = 2$ $\psi = -0.2$
$\chi^2_{1|2} = 1.1 \mid 1.1$ $se_1/se_2 = 2$ $\psi = 0.5$
$\chi^2_{1|2} = 1.3 \mid 1.3$ $se_1/se_2 = 2$ $\psi = 0.5$
$\chi^2_{1|2} = 1.6 \mid 1.6$ $se_1/se_2 = 2$ $\psi = 0.5$
$\chi^2_{1|2} = 1.1 \mid 1.3$ $se_1/se_2 = 2$ $\psi = 0.5$
$\chi^2_{1|2} = 1.1 \mid 1.6$ $se_1/se_2 = 2$ $\psi = 0.5$
$\chi^2_{1|2} = 1.3 \mid 1.6$ $se_1/se_2 = 2$ $\psi = 0.5$
0.32
0.24
0.16
0.08
0
-1
-0.5
0
0.5
1

Supplementary Figure 2. Power comparison between formula (1) and (2) via simulations.

The summary statistics of two GWAS were simulated, each consisting of 50,000 SNPs with 10% true effect, according to the following parameter grid: $\in \{-0.2, 0, 0.5\}$, $se_1^2/se_2^2 \in \{1,4\}$, $\mathrm{E}(\chi^2) \in \{1.1, 1.3, 1.6\}$. For each of the 36 different combinations of parameters, 1,000 replicates were carried out. The x-axis is π and the y-axis is the power. For each configuration, the true effects $\mathrm{E}(\chi^2)$ in the two GWAS is displayed in the top grey bar. For example, $\chi_{1|2}^2 = 1.1|1.1$ means that the true effects of both GWAS are the same with a mean χ^2 value of 1.1. The ratio of se^2 between the two GWAS indicate the reciprocal ratio of sample size. For example, $se_1/se_2 = 1$ means that the two GWAS have the same sample size. The power estimates of formula (2) and formula (1) of two GWAS are indicated in orange and blue dots, respectively. Dots were fitted using local polynomial regression curves.

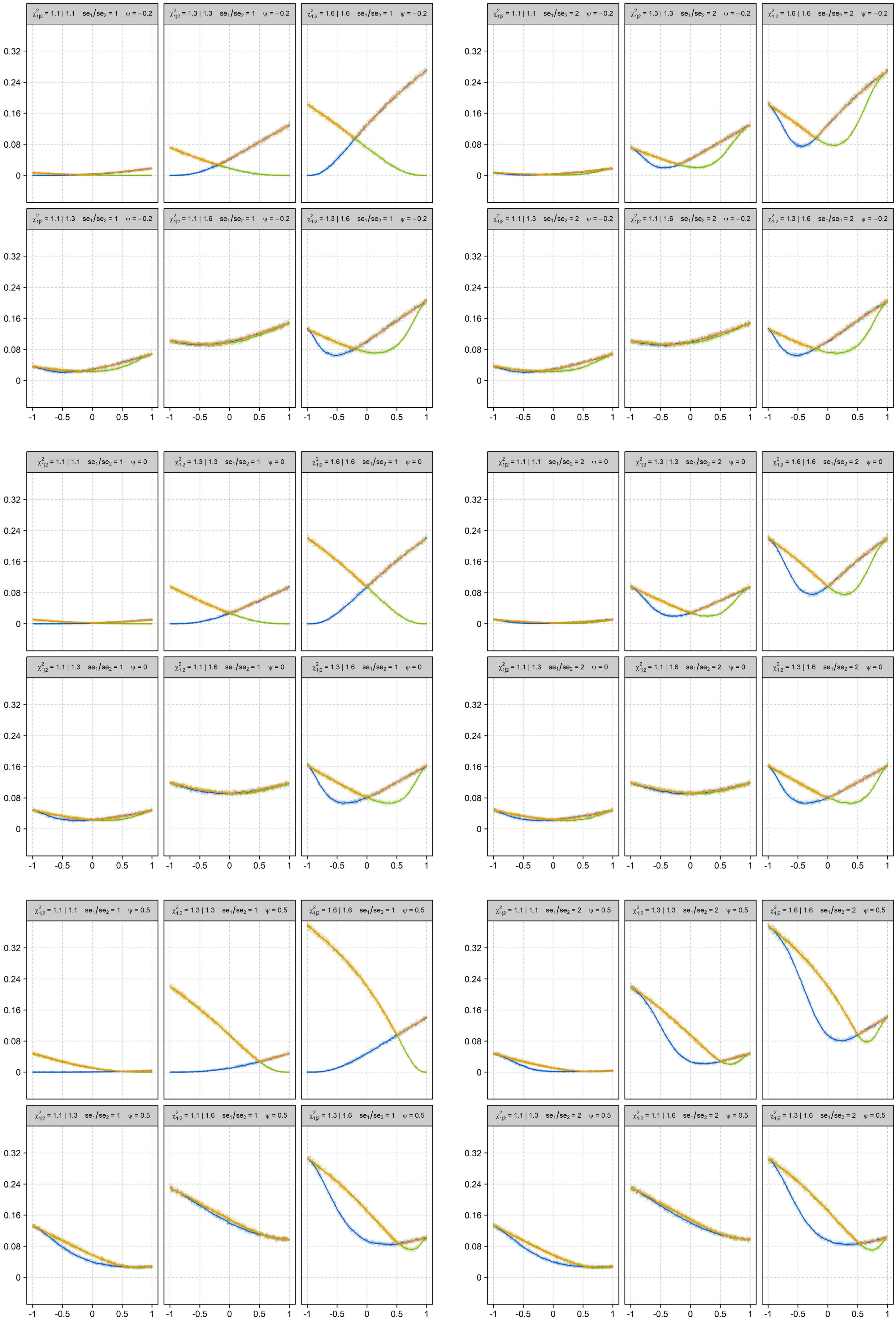

$\chi^2_{1|2}$ = 1.1 | 1.1 se$_1$/se$_2$ = 1 ψ = −0.2
$\chi^2_{1|2}$ = 1.3 | 1.3 se$_1$/se$_2$ = 1 ψ = −0.2
$\chi^2_{1|2}$ = 1.6 | 1.6 se$_1$/se$_2$ = 1 ψ = −0.2
$\chi^2_{1|2}$ = 1.1 | 1.1 se$_1$/se$_2$ = 2 ψ = −0.2
$\chi^2_{1|2}$ = 1.3 | 1.3 se$_1$/se$_2$ = 2 ψ = −0.2
$\chi^2_{1|2}$ = 1.6 | 1.6 se$_1$/se$_2$ = 2 ψ = −0.2
$\chi^2_{1|2}$ = 1.1 | 1.3 se$_1$/se$_2$ = 1 ψ = −0.2
$\chi^2_{1|2}$ = 1.1 | 1.6 se$_1$/se$_2$ = 1 ψ = −0.2
$\chi^2_{1|2}$ = 1.3 | 1.6 se$_1$/se$_2$ = 1 ψ = −0.2
$\chi^2_{1|2}$ = 1.1 | 1.3 se$_1$/se$_2$ = 2 ψ = −0.2
$\chi^2_{1|2}$ = 1.1 | 1.6 se$_1$/se$_2$ = 2 ψ = −0.2
$\chi^2_{1|2}$ = 1.3 | 1.6 se$_1$/se$_2$ = 2 ψ = −0.2
$\chi^2_{1|2}$ = 1.1 | 1.1 se$_1$/se$_2$ = 1 ψ = 0
$\chi^2_{1|2}$ = 1.3 | 1.3 se$_1$/se$_2$ = 1 ψ = 0
$\chi^2_{1|2}$ = 1.6 | 1.6 se$_1$/se$_2$ = 1 ψ = 0
$\chi^2_{1|2}$ = 1.1 | 1.1 se$_1$/se$_2$ = 2 ψ = 0
$\chi^2_{1|2}$ = 1.3 | 1.3 se$_1$/se$_2$ = 2 ψ = 0
$\chi^2_{1|2}$ = 1.6 | 1.6 se$_1$/se$_2$ = 2 ψ = 0
$\chi^2_{1|2}$ = 1.1 | 1.3 se$_1$/se$_2$ = 1 ψ = 0
$\chi^2_{1|2}$ = 1.1 | 1.6 se$_1$/se$_2$ = 1 ψ = 0
$\chi^2_{1|2}$ = 1.3 | 1.6 se$_1$/se$_2$ = 1 ψ = 0
$\chi^2_{1|2}$ = 1.1 | 1.3 se$_1$/se$_2$ = 2 ψ = 0
$\chi^2_{1|2}$ = 1.1 | 1.6 se$_1$/se$_2$ = 2 ψ = 0
$\chi^2_{1|2}$ = 1.3 | 1.6 se$_1$/se$_2$ = 2 ψ = 0
$\chi^2_{1|2}$ = 1.1 | 1.1 se$_1$/se$_2$ = 1 ψ = 0.5
$\chi^2_{1|2}$ = 1.3 | 1.3 se$_1$/se$_2$ = 1 ψ = 0.5
$\chi^2_{1|2}$ = 1.6 | 1.6 se$_1$/se$_2$ = 1 ψ = 0.5
$\chi^2_{1|2}$ = 1.1 | 1.1 se$_1$/se$_2$ = 2 ψ = 0.5
$\chi^2_{1|2}$ = 1.3 | 1.3 se$_1$/se$_2$ = 2 ψ = 0.5
$\chi^2_{1|2}$ = 1.6 | 1.6 se$_1$/se$_2$ = 2 ψ = 0.5
$\chi^2_{1|2}$ = 1.1 | 1.3 se$_1$/se$_2$ = 1 ψ = 0.5
$\chi^2_{1|2}$ = 1.1 | 1.6 se$_1$/se$_2$ = 1 ψ = 0.5
$\chi^2_{1|2}$ = 1.3 | 1.6 se$_1$/se$_2$ = 1 ψ = 0.5
$\chi^2_{1|2}$ = 1.1 | 1.3 se$_1$/se$_2$ = 2 ψ = 0.5
$\chi^2_{1|2}$ = 1.1 | 1.6 se$_1$/se$_2$ = 2 ψ = 0.5
$\chi^2_{1|2}$ = 1.3 | 1.6 se$_1$/se$_2$ = 2 ψ = 0.5
0.32
0.24
0.16
0.08
0
-1
-0.5
0
0.5
1

Supplementary Figure 3. Power comparison between formula (2) and (3) via simulations.

The summary statistics of two GWAS were simulated, each consisting of 50,000 SNPs with 10% true effect, according to the following parameter grid: $\in \{-0.2, 0, 0.5\}$, $se_1^2/se_2^2 \in \{1,4\}$, $\mathrm{E}(\chi^2) \in \{1.1, 1.3, 1.6\}$. For each of the 36 different combinations of parameters, 1,000 replicates were carried out. The x-axis is π and the y-axis is the power. Top grey bar showed the configuration of each simulated scenario, as used in Supplementary Fig. 2. The power estimates of (3), (2) and (2) with negative sign directions of two GWAS are indicated in orange, blue and green dots, respectively. Dots were fitted using local polynomial regression curves.

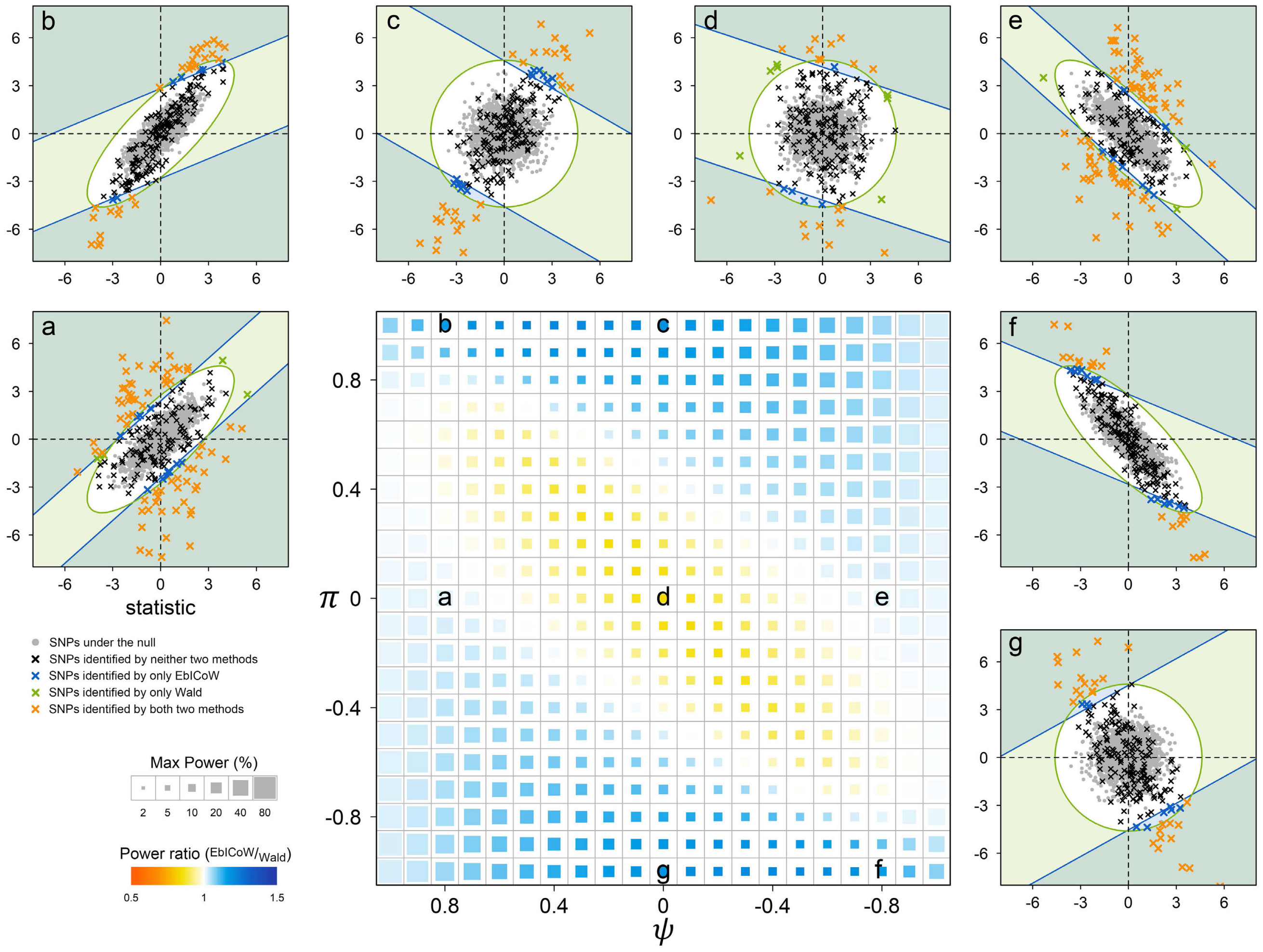

Supplementary Figure 4. Power comparison between EbICoW and Wald test for combining two GWASs with different true effects via simulations.

The summary statistics of two GWASs with different true effect configuration, i.e., $E(\chi_1^2) = 1.2$ and $E(\chi_2^2) = 1.6$, were simulated, each consisting of 50,000 SNPs with 10% true effect, according to the following parameter grid: ψ and $\pi \in \{-0.98, -0.9, -0.8, \ldots, -0.1, 0, 0.1, \ldots, 0.8, 0.9, 0.98\}$. For each of the 441 (21×21) different combinations of π and ψ, 1000 replicates were carried out. The power relationship between EbICoW and Wald is illustrated using a heat map in the center panel. The maximum power of the two methods is proportional to the block size in

the heat map. The power ratio of EbICoW and Wald is expressed by color from red to blue. Taking seven specific combinations of π and ψ as examples, we show in detail the performance of EbICoW and Wald in combining two GWASs (panels a-g surrounding the central panel). For illustration purposes, each surrounding panel is a scatter plot of the test statistics from the two GWASs consisting of 2,000 SNPs with 50% increased true effect compared with the corresponding cell in the heat map, in which the null SNPs are indicated in gray dots and the SNPs with true effects are indicated in colored crosses. Different colors are used to distinguish the significance of EbICoW and Wald, i.e., orange for both significant, blue for EbICoW only significant, green for Wald only significant, and black for non-significant results. The areas outside of the blue lines are the detection range of EbICoW and the area outside of the green ellipse is the detection range of Wald.

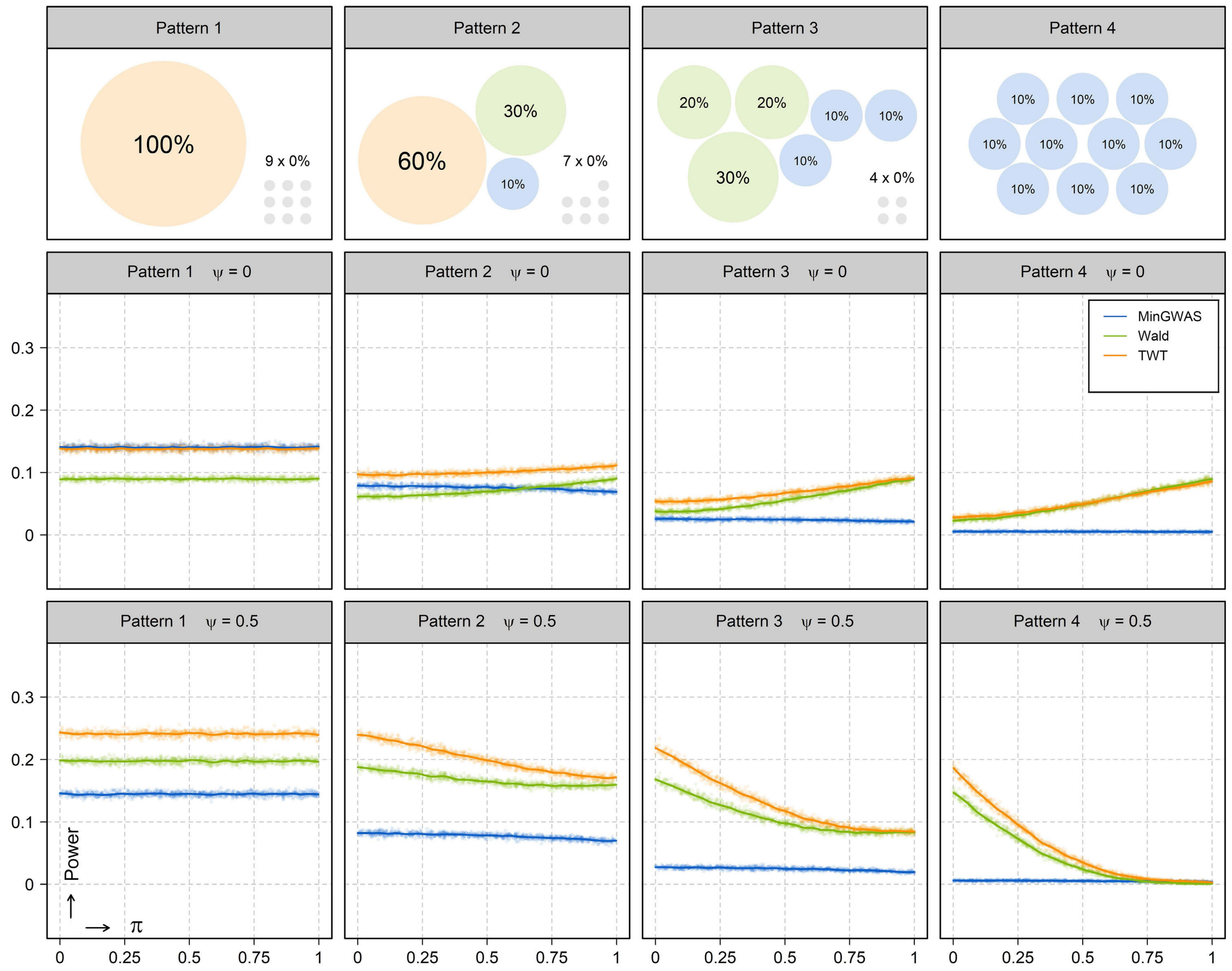

Supplementary Figure 5. Power comparison between Wald test and TWT via simulations.

The summary statistics of ten GWAS were simulated, each consisting of 50,000 SNPs with 10% true effect, according to the two $\psi \in \{0, 0.5\}$ and four patterns of true effect. The configurations of these patterns are the same as in Fig. 2. For each of the 8 different combinations of ψ and pattern of true effect, 1,000 replicates were carried out. The x-axis is π and the y-axis is the power. Each column has the same configuration of the true effects and each row has the same ψ. The power estimates of TWT, Wald, and the MinGWAS of ten GWAS are indicated in orange, green, and blue dots, respectively. Dots were fitted using local polynomial regression curves. Note that the p-values from TWT and the MinGWAS of ten GWAS were additionally adjusted to have the same α as Wald.

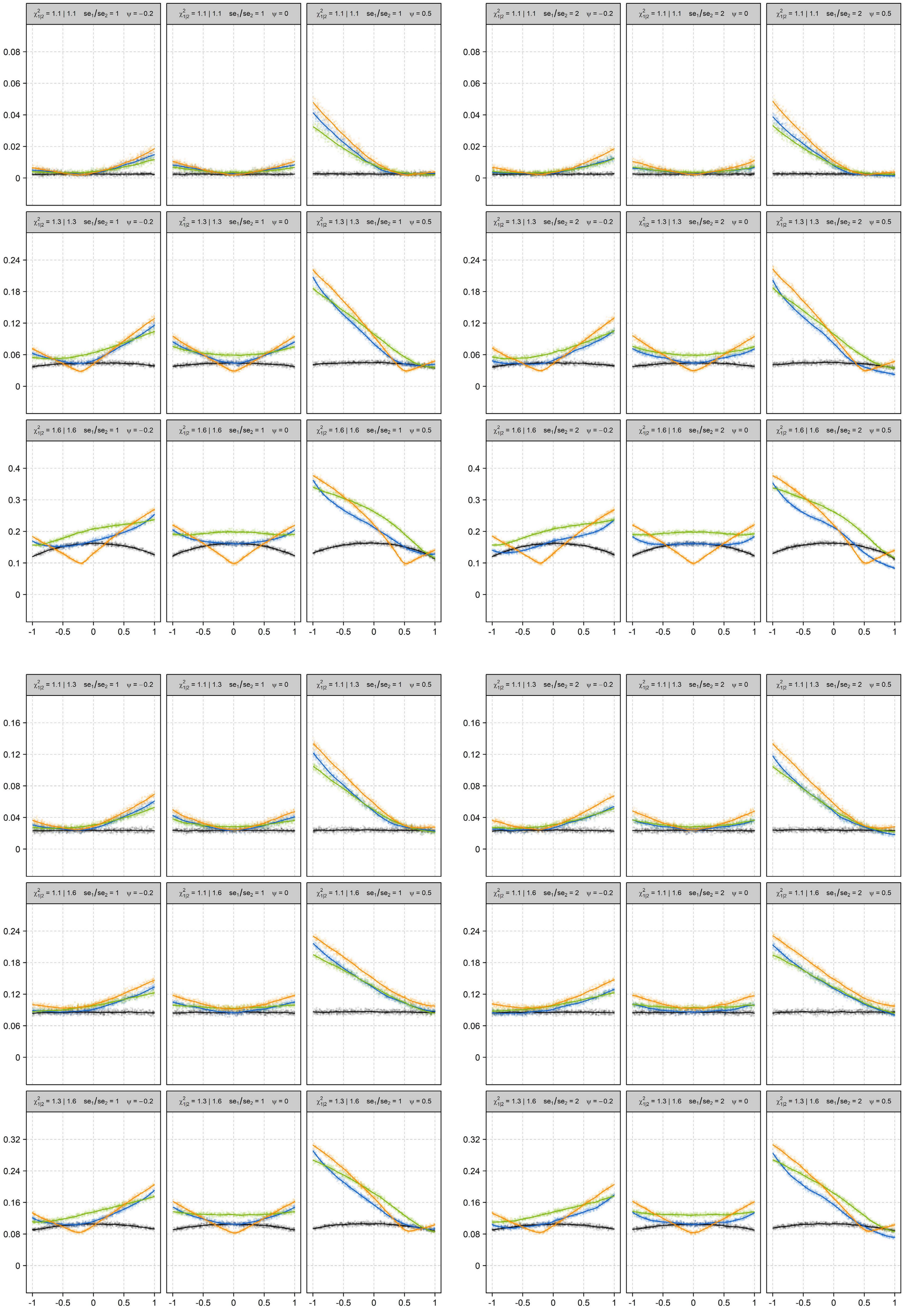

χ²1|2 = 1.1 | 1.1 se1/se2 = 1 ψ = −0.2
χ²1|2 = 1.1 | 1.1 se1/se2 = 1 ψ = 0
χ²1|2 = 1.1 | 1.1 se1/se2 = 1 ψ = 0.5
χ²1|2 = 1.1 | 1.1 se1/se2 = 2 ψ = −0.2
χ²1|2 = 1.1 | 1.1 se1/se2 = 2 ψ = 0
χ²1|2 = 1.1 | 1.1 se1/se2 = 2 ψ = 0.5
χ²1|2 = 1.3 | 1.3 se1/se2 = 1 ψ = −0.2
χ²1|2 = 1.3 | 1.3 se1/se2 = 1 ψ = 0
χ²1|2 = 1.3 | 1.3 se1/se2 = 1 ψ = 0.5
χ²1|2 = 1.3 | 1.3 se1/se2 = 2 ψ = −0.2
χ²1|2 = 1.3 | 1.3 se1/se2 = 2 ψ = 0
χ²1|2 = 1.3 | 1.3 se1/se2 = 2 ψ = 0.5
χ²1|2 = 1.6 | 1.6 se1/se2 = 1 ψ = −0.2
χ²1|2 = 1.6 | 1.6 se1/se2 = 1 ψ = 0
χ²1|2 = 1.6 | 1.6 se1/se2 = 1 ψ = 0.5
χ²1|2 = 1.6 | 1.6 se1/se2 = 2 ψ = −0.2
χ²1|2 = 1.6 | 1.6 se1/se2 = 2 ψ = 0
χ²1|2 = 1.6 | 1.6 se1/se2 = 2 ψ = 0.5
χ²1|2 = 1.1 | 1.3 se1/se2 = 1 ψ = −0.2
χ²1|2 = 1.1 | 1.3 se1/se2 = 1 ψ = 0
χ²1|2 = 1.1 | 1.3 se1/se2 = 1 ψ = 0.5
χ²1|2 = 1.1 | 1.3 se1/se2 = 2 ψ = −0.2
χ²1|2 = 1.1 | 1.3 se1/se2 = 2 ψ = 0
χ²1|2 = 1.1 | 1.3 se1/se2 = 2 ψ = 0.5
χ²1|2 = 1.1 | 1.6 se1/se2 = 1 ψ = −0.2
χ²1|2 = 1.1 | 1.6 se1/se2 = 1 ψ = 0
χ²1|2 = 1.1 | 1.6 se1/se2 = 1 ψ = 0.5
χ²1|2 = 1.1 | 1.6 se1/se2 = 2 ψ = −0.2
χ²1|2 = 1.1 | 1.6 se1/se2 = 2 ψ = 0
χ²1|2 = 1.1 | 1.6 se1/se2 = 2 ψ = 0.5
χ²1|2 = 1.3 | 1.6 se1/se2 = 1 ψ = −0.2
χ²1|2 = 1.3 | 1.6 se1/se2 = 1 ψ = 0
χ²1|2 = 1.3 | 1.6 se1/se2 = 1 ψ = 0.5
χ²1|2 = 1.3 | 1.6 se1/se2 = 2 ψ = −0.2
χ²1|2 = 1.3 | 1.6 se1/se2 = 2 ψ = 0
χ²1|2 = 1.3 | 1.6 se1/se2 = 2 ψ = 0.5

Supplementary Figure 6. Power comparison between EbICoW, MTAG and TWT in two GWASs via simulations.

The summary statistics of two GWASs were simulated, each consisting of 50,000 SNPs with 10% true effect, according to the following parameter grid: $\in \{-0.2, 0, 0.5\}$, $se_1^2/se_2^2 \in \{1,4\}$, $\mathrm{E}(\chi^2) \in \{1.1, 1.3, 1.6\}$. For each of the 36 different combinations of parameters, 1000 replicates were carried out. The x-axis is π and the y-axis is the power. Top grey bar showed the configuration of each simulated scenario, as used in Supplementary Fig. 2. The power estimates of EbICoW, TWT, MTAG, and the Min(p) of two GWASs are indicated in orange, green, blue, and black dots, respectively. Dots were fitted using local polynomial regression curves. Note that the p-values from MTAG, TWT and the Min(p) of two GWAS were additionally adjusted to have the same α with EbICoW.

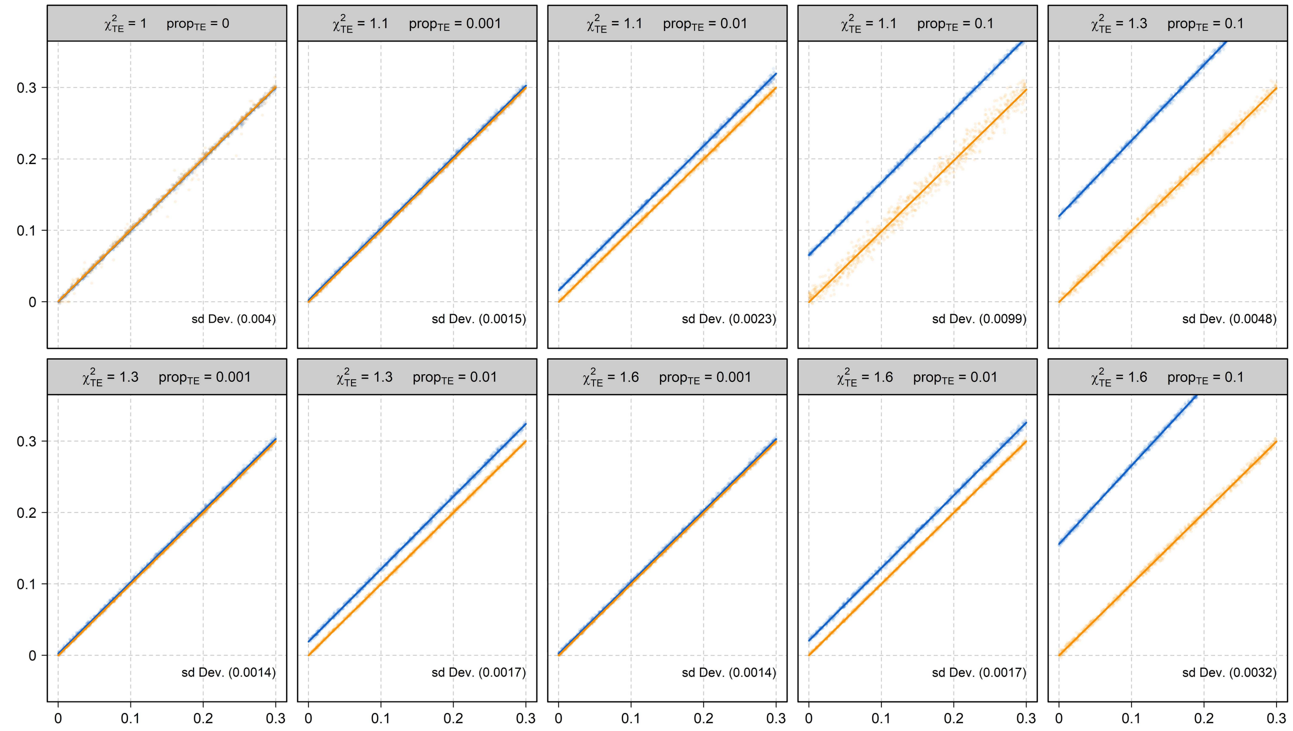

Supplementary Figure 7. Performance of *getI* via simulations.

The test statistics of single trait GWAS were simulated, each consisting of 1,000,000 SNPs, according to the following parameter grid: proportion of SNP with true effect, $prop_{\mathrm{TE}} \in \{0, 0.001, 0.01, 0.1\}$; mean χ^2 of GWAS with true effect, $\mathrm{E}(\chi^2_{\mathrm{TE}}) \in \{1, 1.1, 1.3, 1.6\}$. For each of the 10 different combinations of parameters, 1,000 replicates were carried out. The x-axis is simulated inflation in the form of $\mathrm{E}(\chi^2)$ and the y-axis is the estimated inflation. Each row has the same true effect and each column has the same proportion of SNP with true effect. The inflation estimates of our method and genomic control are indicated in orange and blue dots, respectively. Dots were fitted using lines from linear regression. The standard deviation of difference between simulated inflation and our inflation estimation is showed in right lower corner in each plot.

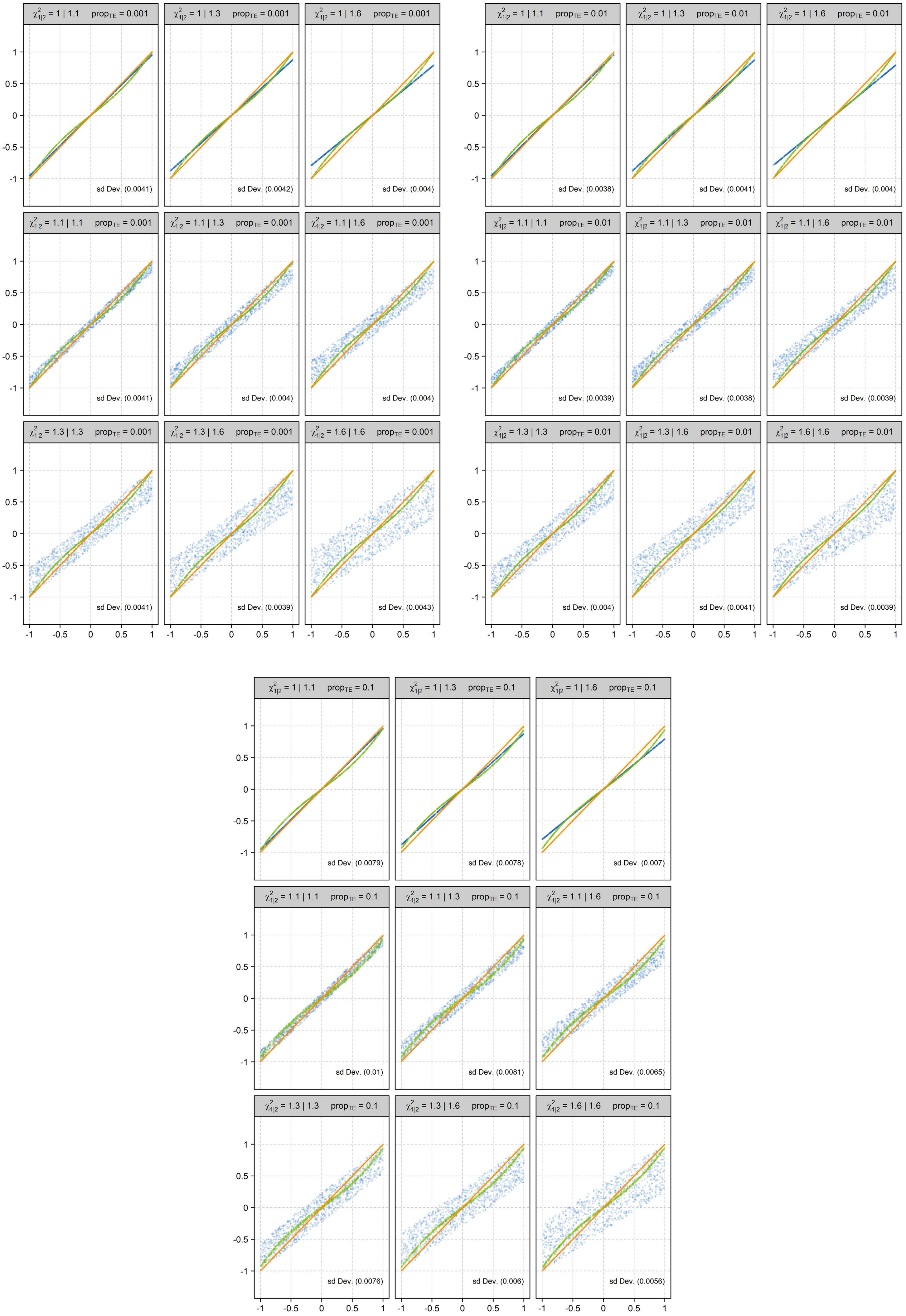
$\chi^2_{1|2}$ = 1 | 1.1 prop$_{TE}$ = 0.001
sd Dev. (0.0041)
$\chi^2_{1|2}$ = 1 | 1.3 prop$_{TE}$ = 0.001
sd Dev. (0.0042)
$\chi^2_{1|2}$ = 1 | 1.6 prop$_{TE}$ = 0.001
sd Dev. (0.004)
$\chi^2_{1|2}$ = 1.1 | 1.1 prop$_{TE}$ = 0.001
sd Dev. (0.0041)
$\chi^2_{1|2}$ = 1.1 | 1.3 prop$_{TE}$ = 0.001
sd Dev. (0.004)
$\chi^2_{1|2}$ = 1.1 | 1.6 prop$_{TE}$ = 0.001
sd Dev. (0.004)
$\chi^2_{1|2}$ = 1.3 | 1.3 prop$_{TE}$ = 0.001
sd Dev. (0.0041)
$\chi^2_{1|2}$ = 1.3 | 1.6 prop$_{TE}$ = 0.001
sd Dev. (0.0039)
$\chi^2_{1|2}$ = 1.6 | 1.6 prop$_{TE}$ = 0.001
sd Dev. (0.0043)
$\chi^2_{1|2}$ = 1 | 1.1 prop$_{TE}$ = 0.01
sd Dev. (0.0038)
$\chi^2_{1|2}$ = 1 | 1.3 prop$_{TE}$ = 0.01
sd Dev. (0.0041)
$\chi^2_{1|2}$ = 1 | 1.6 prop$_{TE}$ = 0.01
sd Dev. (0.004)
$\chi^2_{1|2}$ = 1.1 | 1.1 prop$_{TE}$ = 0.01
sd Dev. (0.0039)
$\chi^2_{1|2}$ = 1.1 | 1.3 prop$_{TE}$ = 0.01
sd Dev. (0.0038)
$\chi^2_{1|2}$ = 1.1 | 1.6 prop$_{TE}$ = 0.01
sd Dev. (0.0039)
$\chi^2_{1|2}$ = 1.3 | 1.3 prop$_{TE}$ = 0.01
sd Dev. (0.004)
$\chi^2_{1|2}$ = 1.3 | 1.6 prop$_{TE}$ = 0.01
sd Dev. (0.0041)
$\chi^2_{1|2}$ = 1.6 | 1.6 prop$_{TE}$ = 0.01
sd Dev. (0.0039)
$\chi^2_{1|2}$ = 1 | 1.1 prop$_{TE}$ = 0.1
sd Dev. (0.0079)
$\chi^2_{1|2}$ = 1 | 1.3 prop$_{TE}$ = 0.1
sd Dev. (0.0078)
$\chi^2_{1|2}$ = 1 | 1.6 prop$_{TE}$ = 0.1
sd Dev. (0.007)
$\chi^2_{1|2}$ = 1.1 | 1.1 prop$_{TE}$ = 0.1
sd Dev. (0.01)
$\chi^2_{1|2}$ = 1.1 | 1.3 prop$_{TE}$ = 0.1
sd Dev. (0.0081)
$\chi^2_{1|2}$ = 1.1 | 1.6 prop$_{TE}$ = 0.1
sd Dev. (0.0065)
$\chi^2_{1|2}$ = 1.3 | 1.3 prop$_{TE}$ = 0.1
sd Dev. (0.0076)
$\chi^2_{1|2}$ = 1.3 | 1.6 prop$_{TE}$ = 0.1
sd Dev. (0.006)
$\chi^2_{1|2}$ = 1.6 | 1.6 prop$_{TE}$ = 0.1
sd Dev. (0.0056)

Supplementary Figure 8. Performance of *getPsi* via simulations.

The test statistics of two GWASs were simulated, each consisting of 1,000,000 SNPs with randomly preset $\pi \in (-1, 1)$, according to the following parameter grid: proportion of SNPs with true effect, $prop_{\mathrm{TE}} \in \{0.001, 0.01, 0.1\}$, and $\mathrm{E}(\chi^2) \in \{1, 1.1, 1.3, 1.6\}$. For example, $\chi^2_{1|2} = 1|1.1$ means that the GWAS 1 is under the null with a mean χ^2 value of 1.0 and GWAS 2 has true effects with a mean χ^2 value of 1.1. For each of the 27 different combinations of parameters, 1,000 replicates were carried out. The x-axis is preset ψ and the y-axis is the estimated ψ. Our method (orange dots) is compared with another two different methods for estimating ψ. One is to simply compute the correlation between two GWASs using all test statistics (blue dots). Another is to compute the correlation between two GWASs but only focusing on those nominally non-significant SNPs (green dots). The standard deviation of difference between the simulated ψ and estimated ψ based on our method is displayed in right lower corner in each plot.

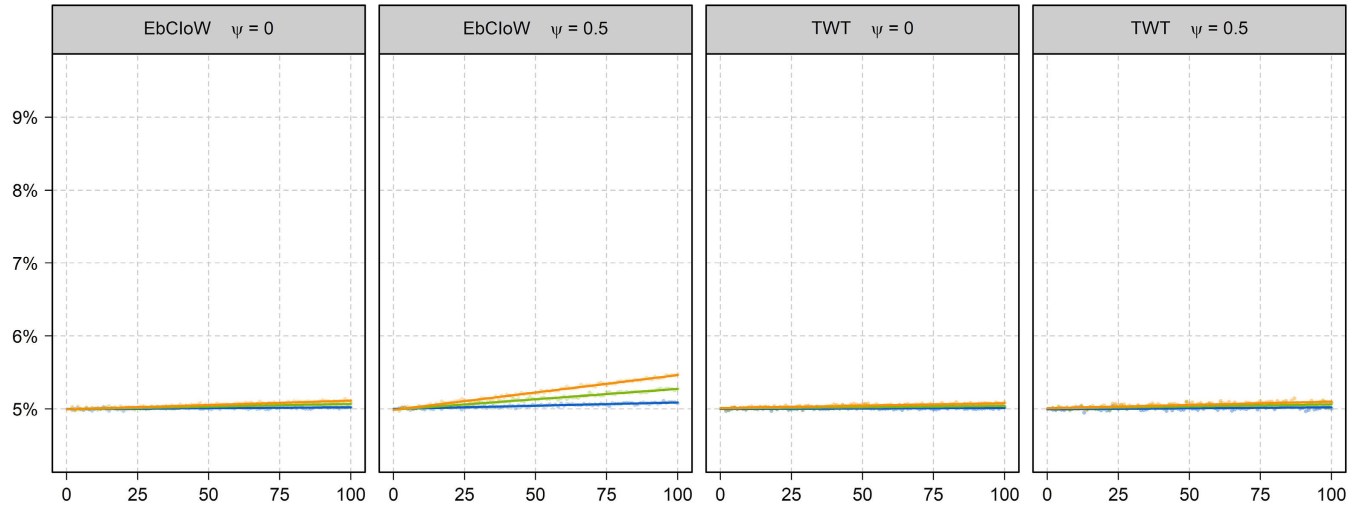

Supplementary Figure 9. Type I error rate of EbICoW and TWT

To compare the α of EbICoW and TWT in presence of estimation error from *getI* and *getPsi*, we simulate test statistics of multiple GWASs, each consisting of 10,000 SNPs, with the parameter grid: GWAS number $K \in \{2, 3, \dots, 100\}$, $\psi \in \{0, 0.5\}$, and randomly noise added to s and ψ with three descending levels (large, orange; medium, green; small, blue). The x-axis is the GWAS number, and the y-axis is the α. 1,000 replicates were carried out for all scenarios. Dots were fitted using lines from linear regression.

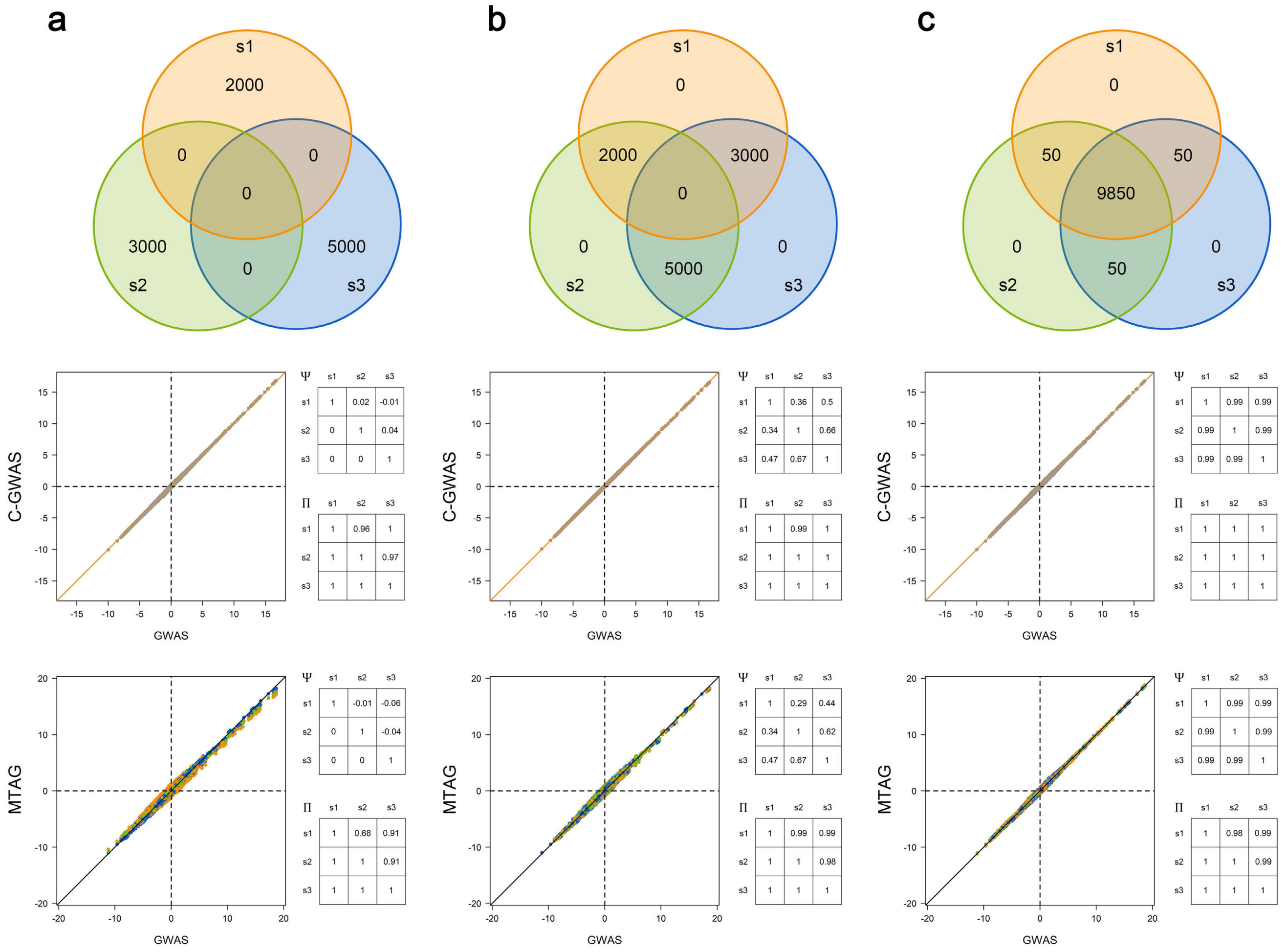

Supplementary Figure 10. Performance of C–GWAS and MTAG in combining GWASs in overlapping samples.

Based on the real genotype data of 10,000 participants from the Rotterdam Study (526,822 SNPs of chromosome 1), a normally distributed variable was simulated as the only phenotype in such a way that 10% of the phenotypic variance is explained by 1% of all SNPs, whose true effects follow a normal distribution. Three sub-datasets were created with different sample overlapping (upper panel): (**a**) non-overlapping with sizes of 2000, 3000, and 5000; (**b**) partially overlapping with sizes of 5000, 7000, and 8000; and (**c**) almost complete overlapping of 9950 each. C-

GWAS and MTAG were conducted to combine three GWASs in sub-datasets and a GWAS was conducted in all samples. Test statistics of all SNPs from the C-GWAS and three MTAG results are plotted against those from the GWAS (grey points of C-GWAS, middle panel; orange, green and blue points of MTAG, lower panel). Orange lines indicate the equality of test statistics in C-GWAS and GWAS. Black lines indicate the equality of test statistics in MTAG and GWAS. Expected and estimated background and effect correlation (left lower and right upper part of each matrix) of three configurations from C-GWAS and MTAG are illustrated at right of each scatter-plots. Noted that the expected and estimated $\mathbf{\Psi}$ and $\mathbf{\Pi}$ from MTAG was obtained by scaling the covariance matrix $\mathbf{\Sigma}$ and $\mathbf{\Omega}$ estimated in MTAG.

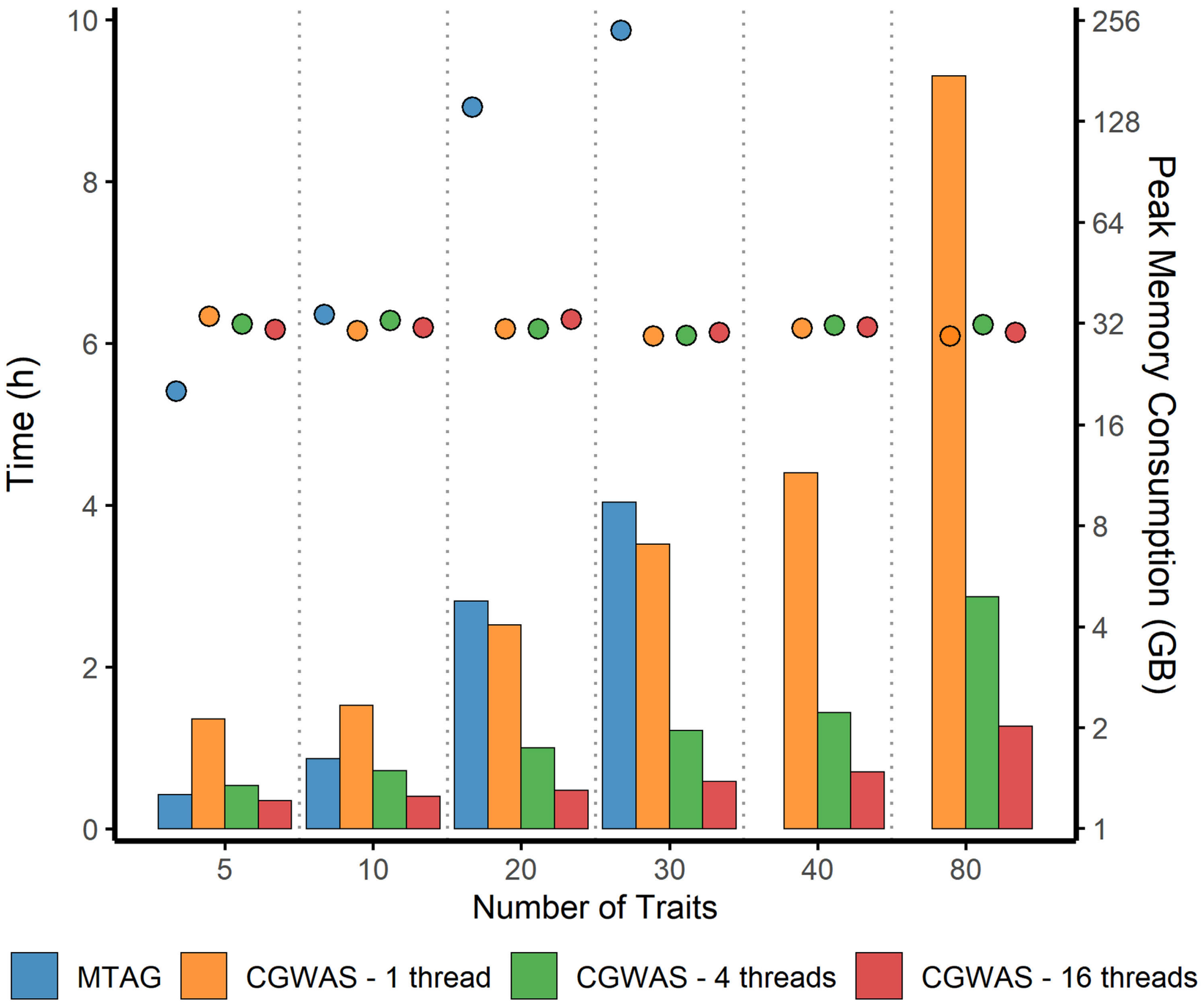

Supplementary Figure 11. The computational efficiency of C–GWAS and MTAG. We assessed the performance of C-GWAS and MTAG on a machine with 72 cores Intel Xeon CPU at 2.30GHz and 256GB RAM, using simulated data (6 million SNPs; 5, 10, 20, 30, 40, and 80 GWASs; 1, 2, 4, 8, and 16 paralleled threads, see methods). Scenarios in each configuration were replicated testing for 3 times, and the mean performance was reported. Bar plots with colors indicate the computational times (left y-axis) of MTAG or C-GWAS with different number of paralleled threads. Plots connected by lines in colors indicate peak memory (GB, right y-axis) used of MTAG or C-GWAS. Note that MTAG failed in running analysis of 40 and 80 GWASs.

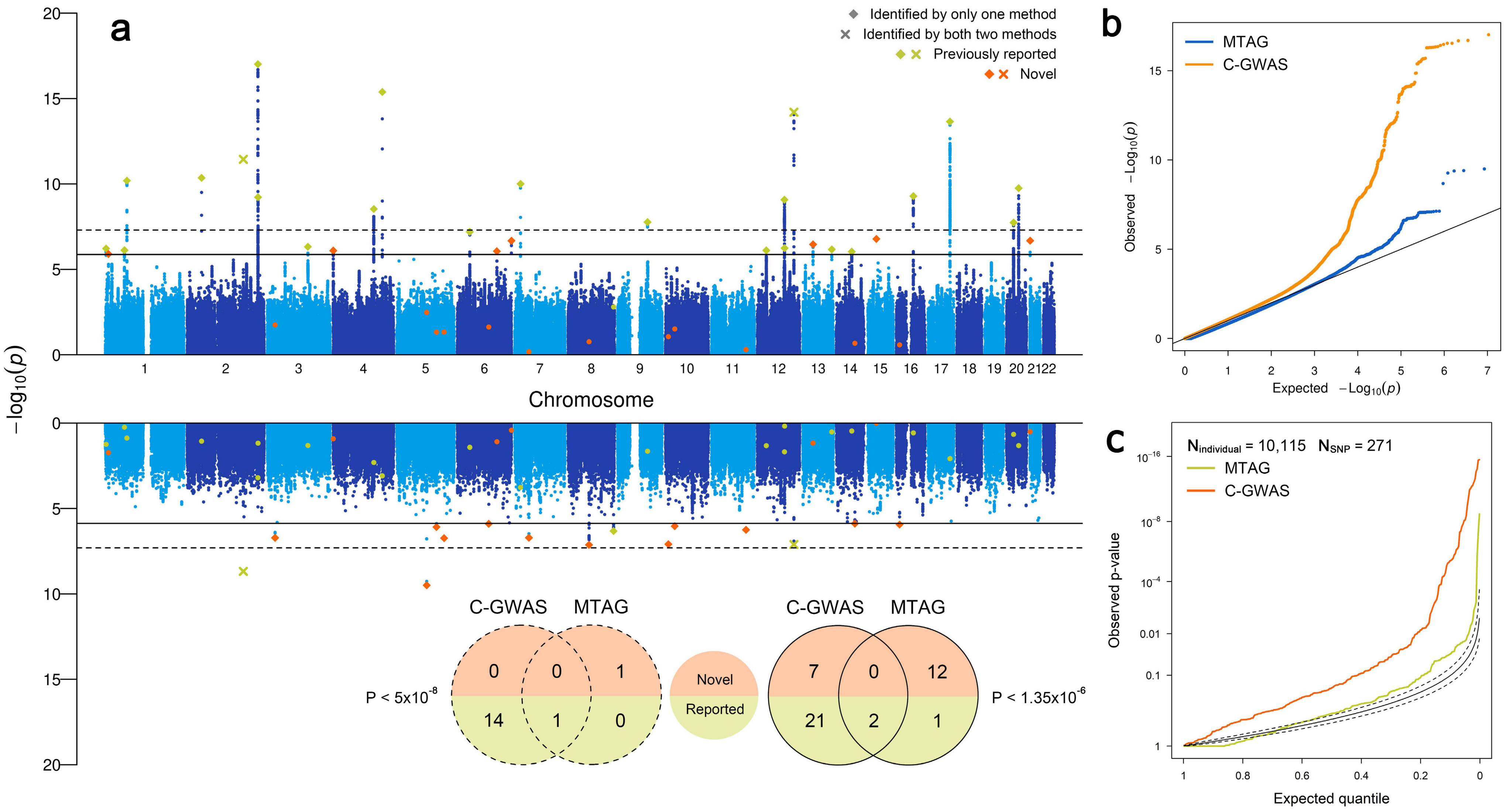

a
Identified by only one method
Identified by both two methods
Previously reported
Novel
−log10(p)
Chromosome
C-GWAS
MTAG
P < 5x10-8
Novel
Reported
P < 1.35x10-6
b
MTAG
C-GWAS
Observed −Log10(p)
Expected −Log10(p)
c
Nindividual = 10,115 NSNP = 271
MTAG
C-GWAS
Observed p-value
Expected quantile

Supplementary Figure 12. Comparison of C–GWAS and MTAG using 30 facial phenotypes.

Because conducting MTAG analysis for 78 facial phenotypes exceeded our computational capacity, we focused on 30 phenotypes on the left side of the face. C-GWAS and MTAG analyses were carried out using GWAS summary statistics generated from the discovery sample consisting of 10,115 individuals of European decent. Because MTAG provided one outcome for each trait (total 30 outcomes), the adjusted minimal p-values of 30 MTAG p-values were considered as the final result from MTAG for comparison with C-GWAS. The adjustment was achieved using the *getCoef* function of C-GWAS. The C-GWAS p-values (upper part of **a**) and the MTAG adjusted p-values (lower part of **a**) are plotted using a Miami plot (**a**) and a Q-Q plot (**b**). The study-wide significance threshold ($p = 5 \times 10^{-8}$) is indicated using dashed lines and the study-wide suggestive significance threshold ($p = 1.35 \times 10^{-6}$) is indicated using solid lines. All regional lead SNPs passing the suggestive line are highlighted using different shapes (cross for significant in both C-GWAS and MTAG; diamond for significant only in one method) in plots of identified method and colors (previous known face-associated loci are indicated in green and novel loci are indicated in orange) in plots of both C-GWAS and MTAG. The logical relationship between different sets of loci is illustrated using a Venn diagram (green for known loci and orange for novel loci). A list of 271 SNPs previously associated with facial variation was looked up in the C-GWAS and GWAS results of the discovery sample (n=10,115, **c**).

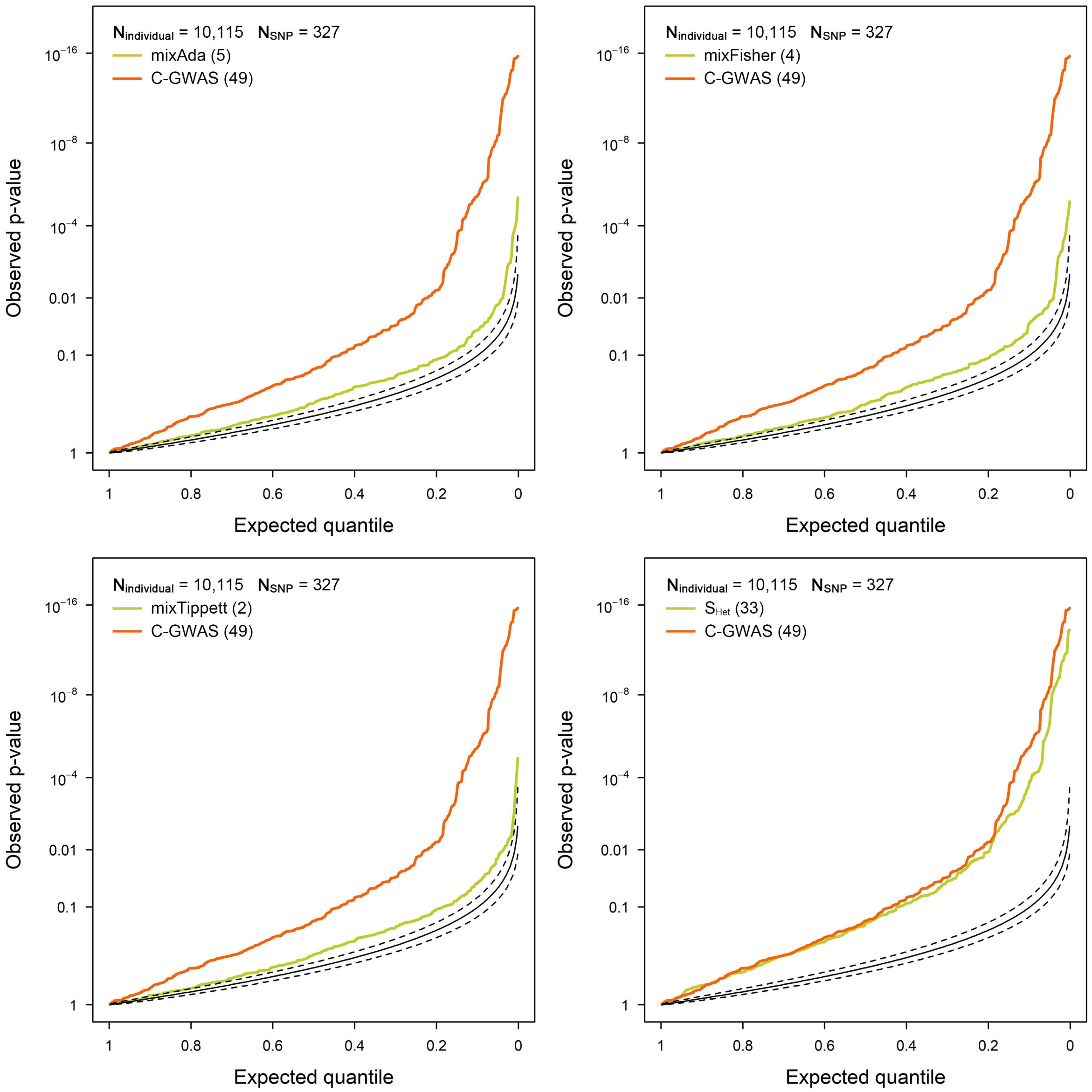

Supplementary Figure 13. Comparison of C-GWAS, mixAda, mixFisher, mixTippett and S_{Het} using previously established face-associated SNPs.

A list of 327 previously established face-associated SNPs was looked up in the C-GWAS in comparison with mixAda, mixFisher, mixTippett and S_{Het}. The number of significant SNPs passing Bonferroni correction was displayed behind the legend of each method. Solid and dashed lines denote the expected null with 95% CI.

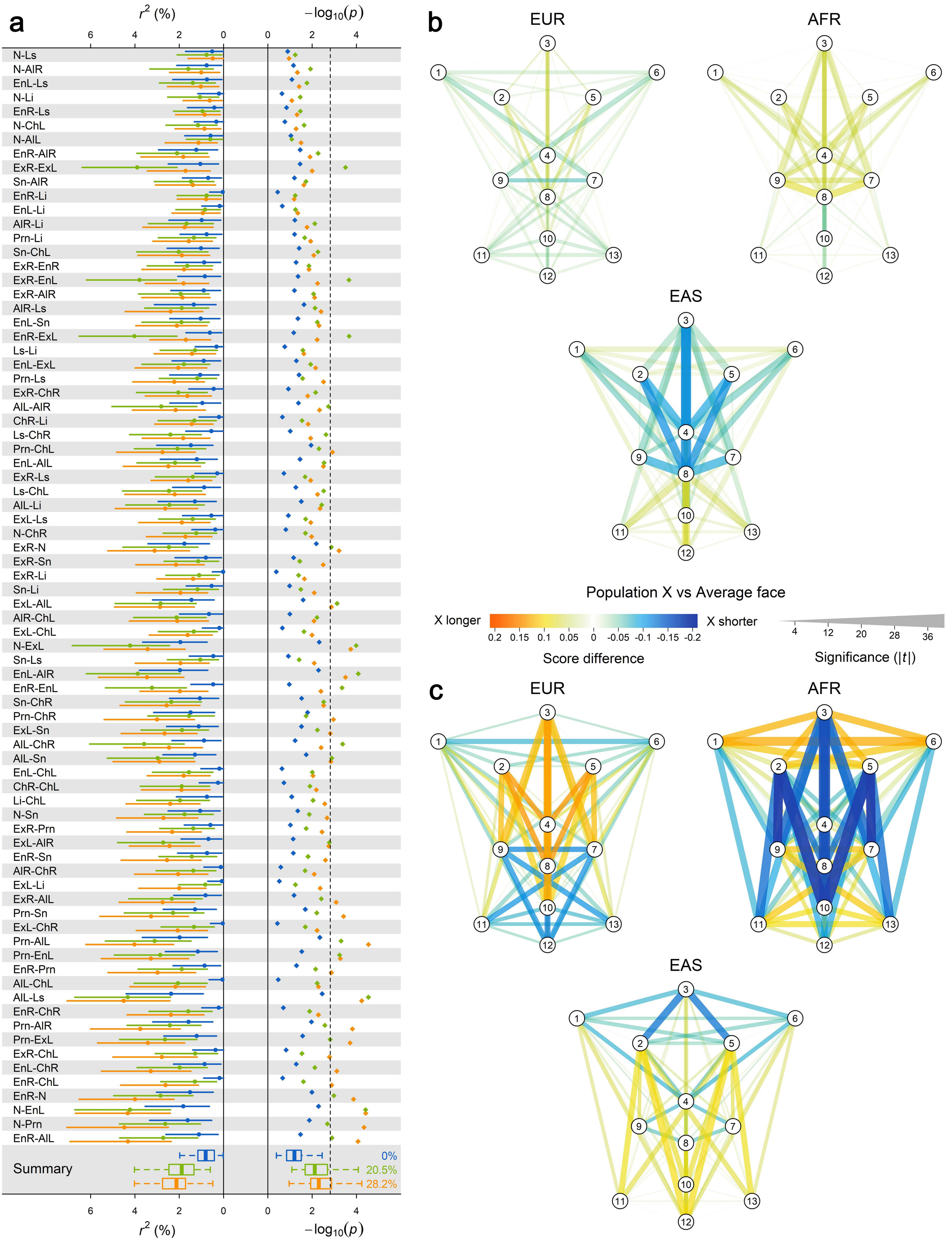
a
r^2 (%)
$-\log_{10}(p)$
N-Ls
N-AlR
EnL-Ls
N-Li
EnR-Ls
N-ChL
N-AlL
EnR-AlR
ExR-ExL
Sn-AlR
EnR-Li
EnL-Li
AlR-Li
Prn-Li
Sn-ChL
ExR-EnR
ExR-EnL
ExR-AlR
AlR-Ls
EnL-Sn
EnR-ExL
Ls-Li
EnL-ExL
Prn-Ls
ExR-ChR
AlL-AlR
ChR-Li
Ls-ChR
Prn-ChL
EnL-AlL
ExR-Ls
Ls-ChL
AlL-Li
ExL-Ls
N-ChR
ExR-N
ExR-Sn
ExR-Li
Sn-Li
ExL-AlL
AlR-ChL
ExL-ChL
N-ExL
Sn-Ls
EnL-AlR
EnR-EnL
Sn-ChR
Prn-ChR
ExL-Sn
AlL-ChR
AlL-Sn
EnL-ChL
ChR-ChL
Li-ChL
N-Sn
ExR-Prn
ExL-AlR
EnR-Sn
AlR-ChR
ExL-Li
ExR-AlL
Prn-Sn
ExL-ChR
Prn-AlL
Prn-EnL
EnR-Prn
AlL-ChL
AlL-Ls
EnR-ChR
Prn-AlR
Prn-ExL
ExR-ChL
EnL-ChR
EnR-ChL
EnR-N
N-EnL
N-Prn
EnR-AlL
Summary
0%
20.5%
28.2%
b
EUR
AFR
EAS
Population X vs Average face
X longer
X shorter
0.2 0.15 0.1 0.05 0 -0.05 -0.1 -0.15 -0.2
Score difference
4 12 20 28 36
Significance (|t|)
c
EUR
AFR
EAS

Supplementary Figure 14. Polygenic Risk Scores (PRS) derived based on C–GWAS and MinGWAS findings.

(**a**), A PRS analysis was carried out in the replication cohort of 1,174 individuals from the Rotterdam Study (RS). PRSs were separately constructed based on C-GWAS findings (57 lead SNPs) and MinGWAS findings (57 lead SNPs or 17 lead SNPs) for all 78 facial traits. Explained variance in the full samples (r^2, dot) and its 95% confidence intervals (error bar) obtained from 1,000 times randomly sampling are presented on the left side and the significance for testing H0: r^2=0 using the z-test is displayed on the right side (orange for C-GWAS, green for MinGWAS of 57 SNPs and blue for MinGWAS of 17 SNPs). Boxplot below display the distribution of r^2 and z-test significance of 78 facial traits. The band indicates the median, the box indicates the first and third quartiles, and the whiskers indicate 1.5 times of interquartile range from box. The proportion beside right boxplot indicate the percentage of significant p-values from 78 z-tests after Bonferroni correction. (**b**–**c**), PRSs were constructed for 78 facial traits in 1,668 individuals from three major continental groups (Europe, EUR; Africa, AFR; and East Asian, EAS) of the 1000 Genomes Project (1000G). Facial variation explainable by MinGWAS (**b**) and C-GWAS (**c**) PRS was compared with the average PRS across all continents using two-side t-test.

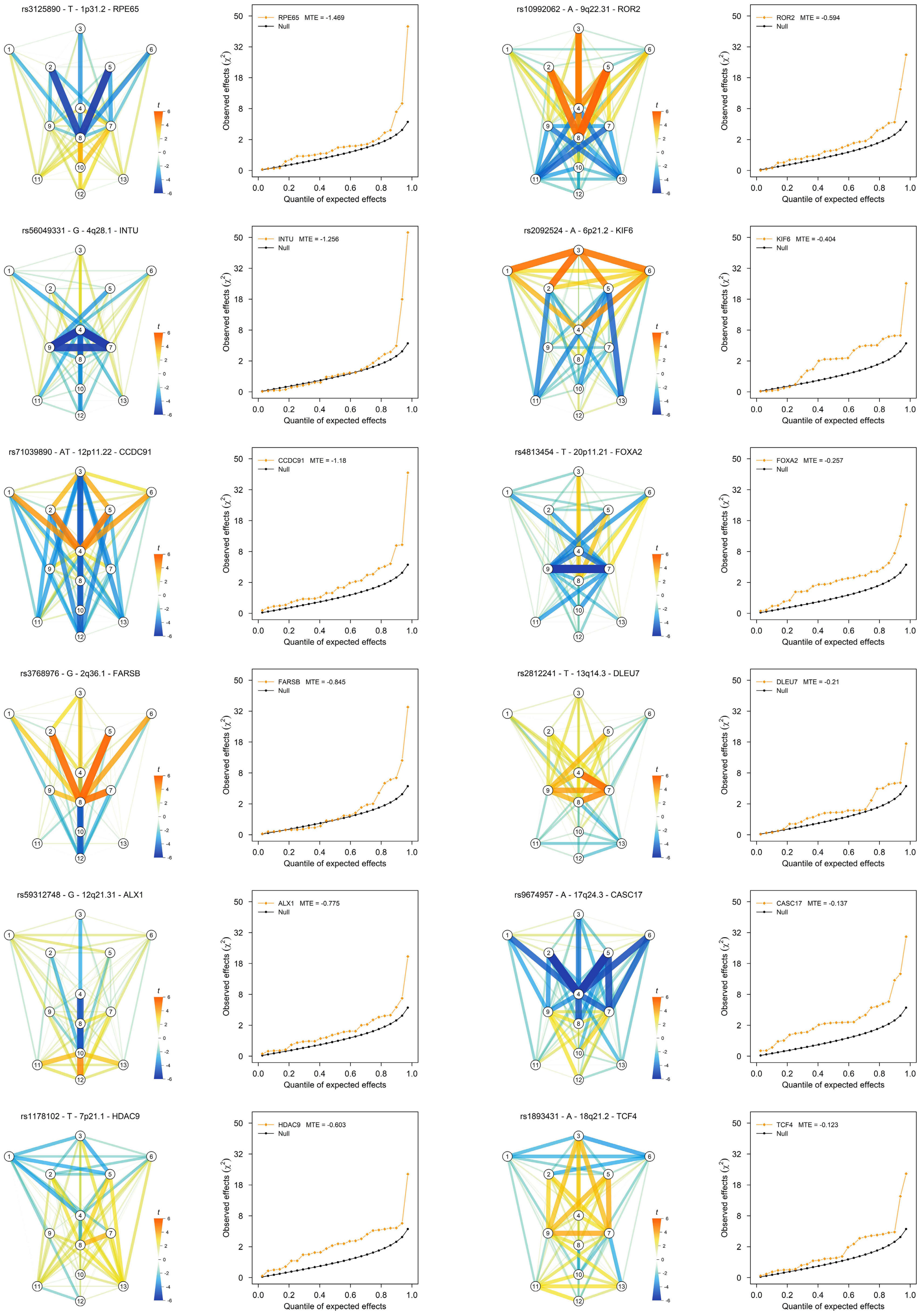
rs3125890 - T - 1p31.2 - RPE65
RPE65 MTE = -1.469
Null
rs10992062 - A - 9q22.31 - ROR2
ROR2 MTE = -0.594
Null
rs56049331 - G - 4q28.1 - INTU
INTU MTE = -1.256
Null
rs2092524 - A - 6p21.2 - KIF6
KIF6 MTE = -0.404
Null
rs71039890 - AT - 12p11.22 - CCDC91
CCDC91 MTE = -1.18
Null
rs4813454 - T - 20p11.21 - FOXA2
FOXA2 MTE = -0.257
Null
rs3768976 - G - 2q36.1 - FARSB
FARSB MTE = -0.845
Null
rs2812241 - T - 13q14.3 - DLEU7
DLEU7 MTE = -0.21
Null
rs59312748 - G - 12q21.31 - ALX1
ALX1 MTE = -0.775
Null
rs9674957 - A - 17q24.3 - CASC17
CASC17 MTE = -0.137
Null
rs1178102 - T - 7p21.1 - HDAC9
HDAC9 MTE = -0.603
Null
rs1893431 - A - 18q21.2 - TCF4
TCF4 MTE = -0.123
Null
Observed effects (χ^2)
Quantile of expected effects
t

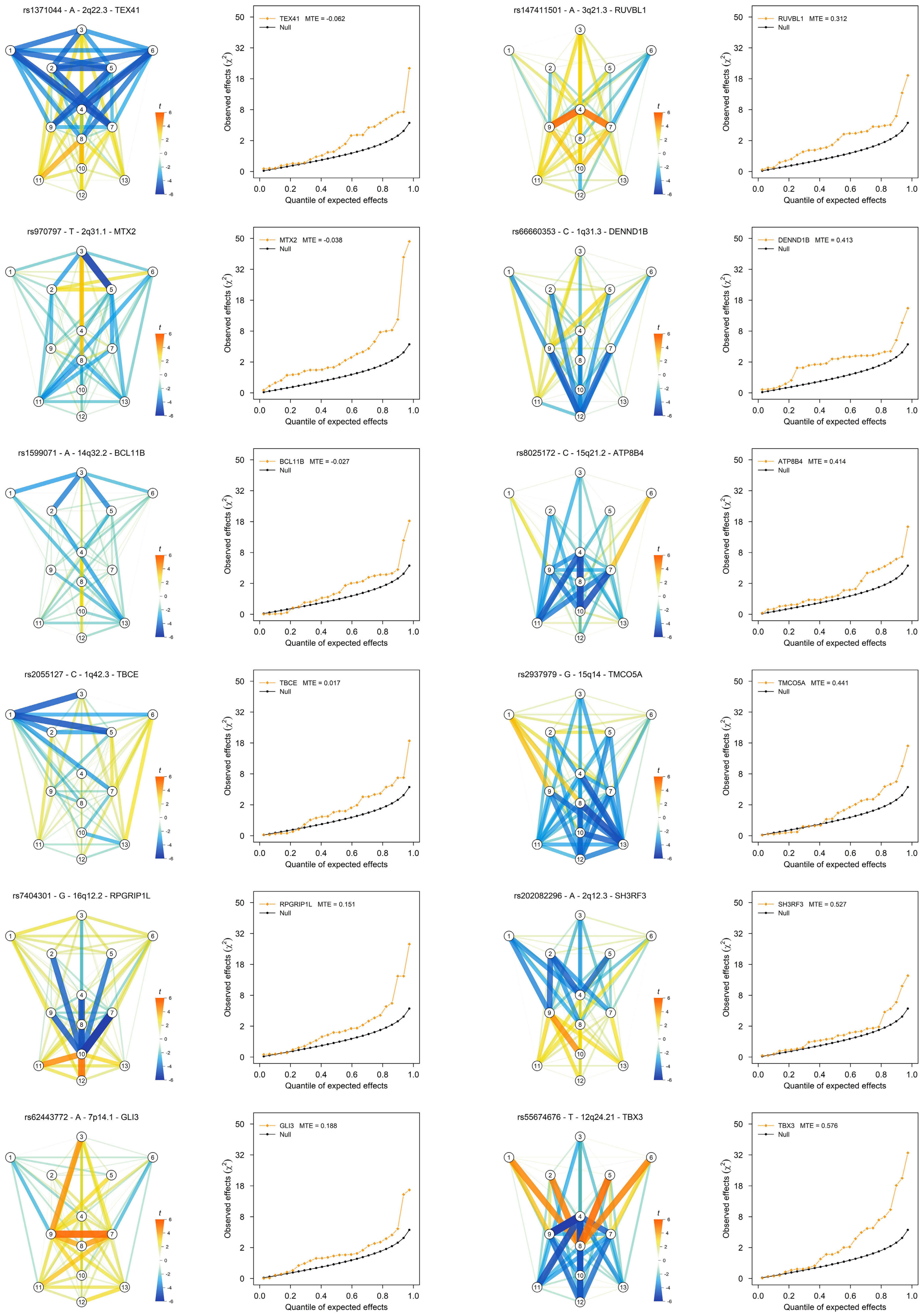
rs1371044 - A - 2q22.3 - TEX41
TEX41 MTE = -0.062
Null
rs147411501 - A - 3q21.3 - RUVBL1
RUVBL1 MTE = 0.312
Null
rs970797 - T - 2q31.1 - MTX2
MTX2 MTE = -0.038
Null
rs66660353 - C - 1q31.3 - DENND1B
DENND1B MTE = 0.413
Null
rs1599071 - A - 14q32.2 - BCL11B
BCL11B MTE = -0.027
Null
rs8025172 - C - 15q21.2 - ATP8B4
ATP8B4 MTE = 0.414
Null
rs2055127 - C - 1q42.3 - TBCE
TBCE MTE = 0.017
Null
rs2937979 - G - 15q14 - TMCO5A
TMCO5A MTE = 0.441
Null
rs7404301 - G - 16q12.2 - RPGRIP1L
RPGRIP1L MTE = 0.151
Null
rs202082296 - A - 2q12.3 - SH3RF3
SH3RF3 MTE = 0.527
Null
rs62443772 - A - 7p14.1 - GLI3
GLI3 MTE = 0.188
Null
rs55674676 - T - 12q24.21 - TBX3
TBX3 MTE = 0.576
Null
Observed effects (χ^2)
Quantile of expected effects
t

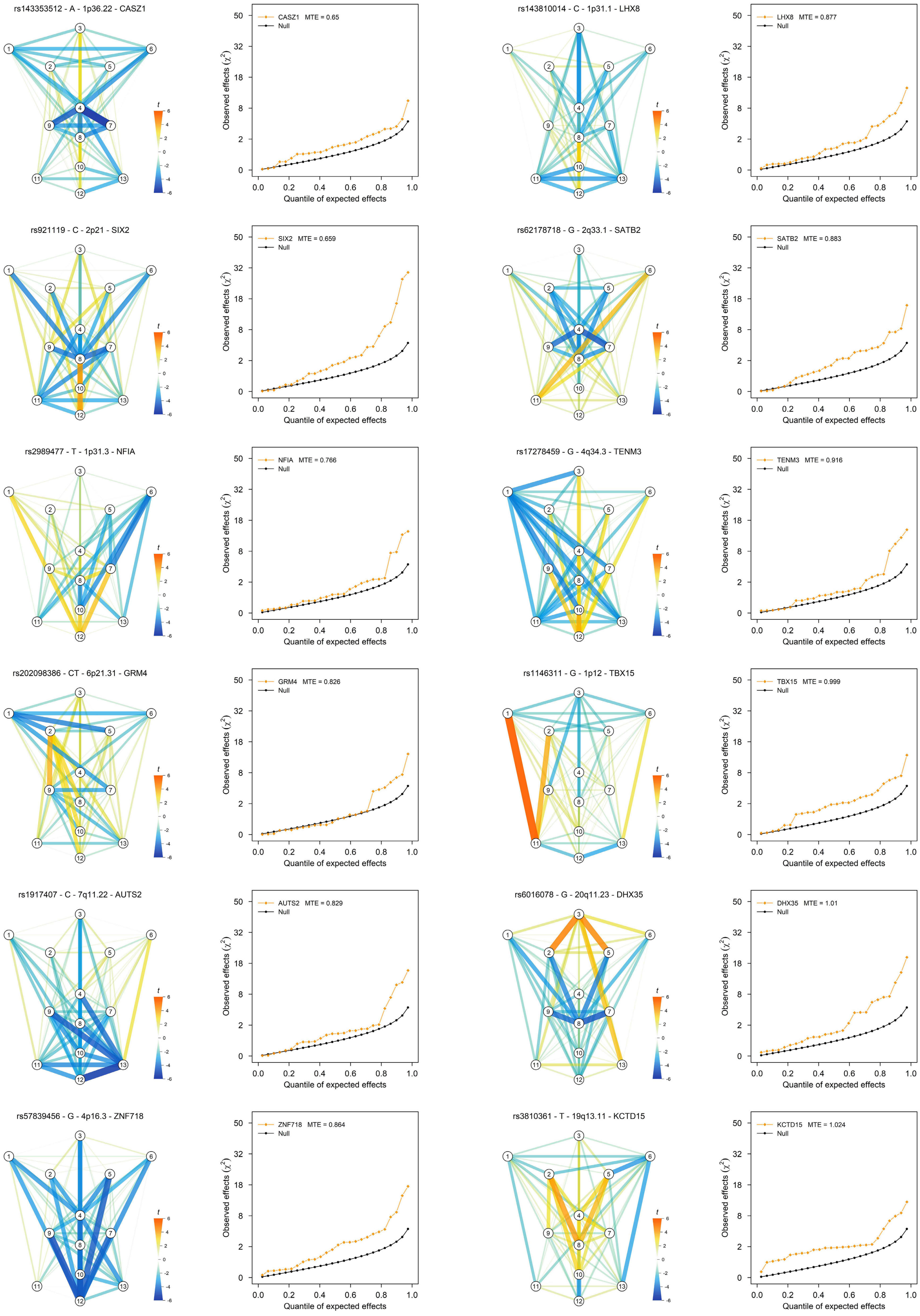

rs143353512 - A - 1p36.22 - CASZ1
CASZ1 MTE = 0.65
Null
rs143810014 - C - 1p31.1 - LHX8
LHX8 MTE = 0.877
Null
rs921119 - C - 2p21 - SIX2
SIX2 MTE = 0.659
Null
rs62178718 - G - 2q33.1 - SATB2
SATB2 MTE = 0.883
Null
rs2989477 - T - 1p31.3 - NFIA
NFIA MTE = 0.766
Null
rs17278459 - G - 4q34.3 - TENM3
TENM3 MTE = 0.916
Null
rs202098386 - CT - 6p21.31 - GRM4
GRM4 MTE = 0.826
Null
rs1146311 - G - 1p12 - TBX15
TBX15 MTE = 0.999
Null
rs1917407 - C - 7q11.22 - AUTS2
AUTS2 MTE = 0.829
Null
rs6016078 - G - 20q11.23 - DHX35
DHX35 MTE = 1.01
Null
rs57839456 - G - 4p16.3 - ZNF718
ZNF718 MTE = 0.864
Null
rs3810361 - T - 19q13.11 - KCTD15
KCTD15 MTE = 1.024
Null
Observed effects (χ^2)
Quantile of expected effects
t

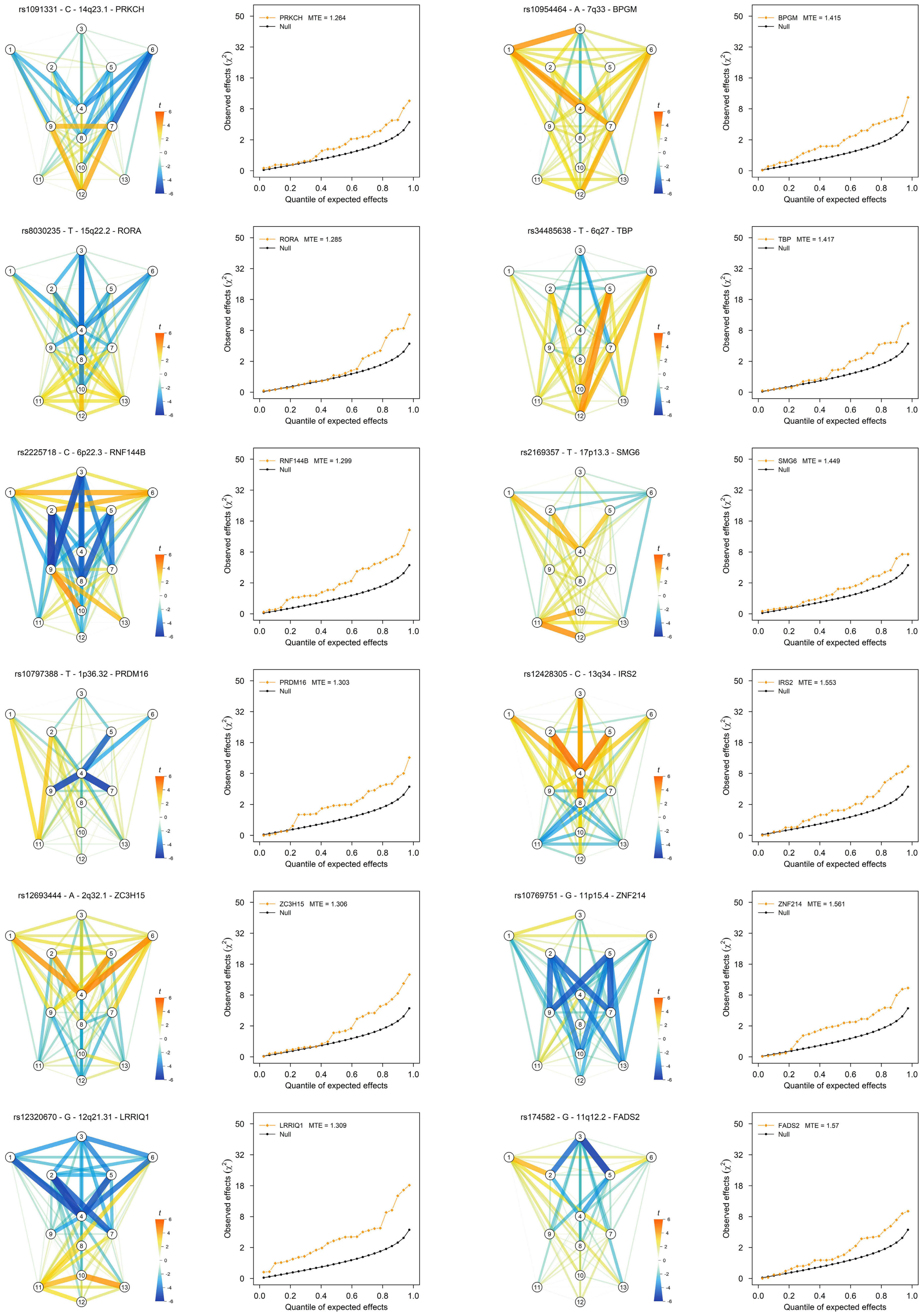

rs1091331 - C - 14q23.1 - PRKCH
PRKCH MTE = 1.264
Null
rs10954464 - A - 7q33 - BPGM
BPGM MTE = 1.415
Null
rs8030235 - T - 15q22.2 - RORA
RORA MTE = 1.285
Null
rs34485638 - T - 6q27 - TBP
TBP MTE = 1.417
Null
rs2225718 - C - 6p22.3 - RNF144B
RNF144B MTE = 1.299
Null
rs2169357 - T - 17p13.3 - SMG6
SMG6 MTE = 1.449
Null
rs10797388 - T - 1p36.32 - PRDM16
PRDM16 MTE = 1.303
Null
rs12428305 - C - 13q34 - IRS2
IRS2 MTE = 1.553
Null
rs12693444 - A - 2q32.1 - ZC3H15
ZC3H15 MTE = 1.306
Null
rs10769751 - G - 11p15.4 - ZNF214
ZNF214 MTE = 1.561
Null
rs12320670 - G - 12q21.31 - LRRIQ1
LRRIQ1 MTE = 1.309
Null
rs174582 - G - 11q12.2 - FADS2
FADS2 MTE = 1.57
Null
t
Observed effects (χ²)
Quantile of expected effects

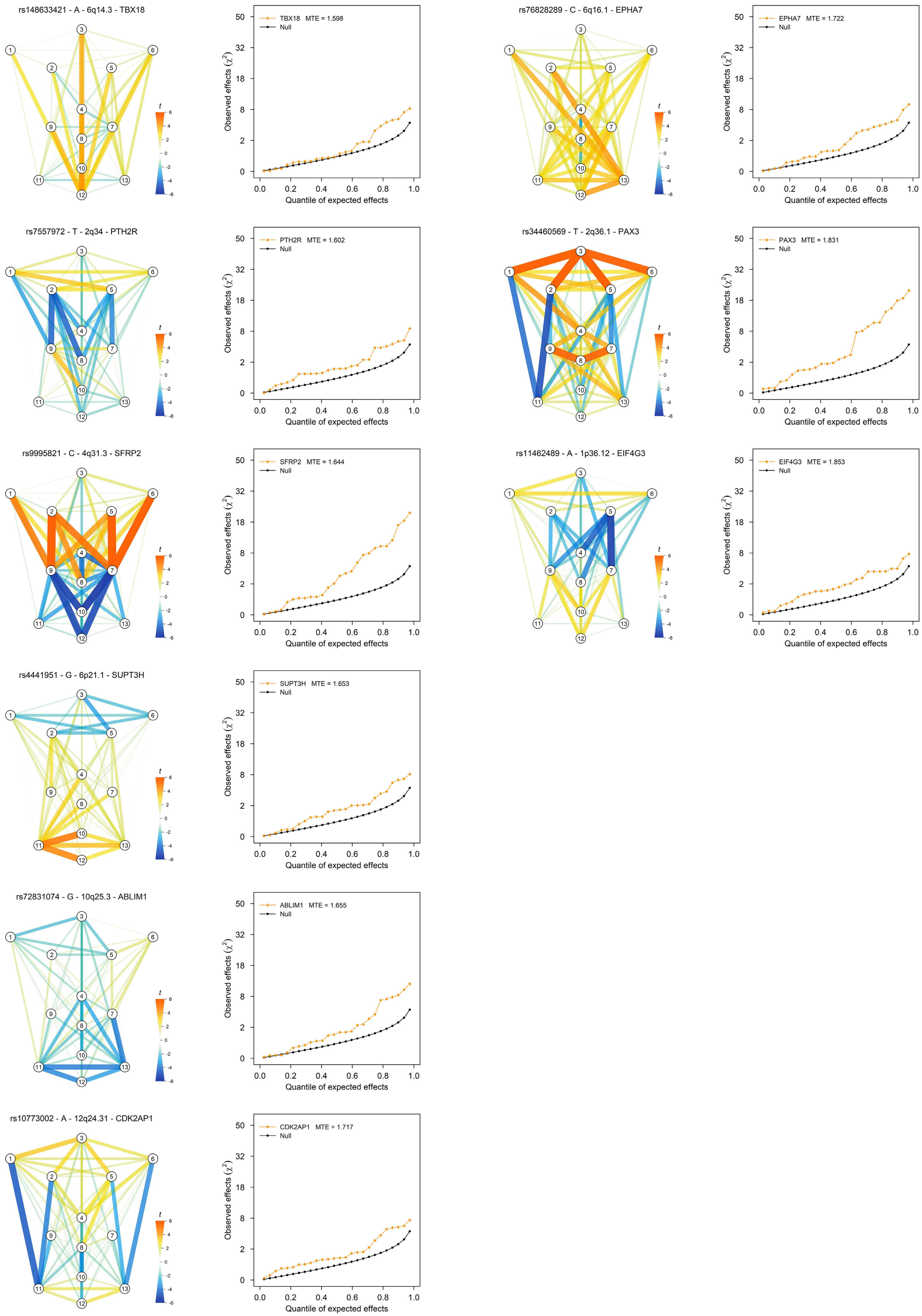

rs148633421 - A - 6q14.3 - TBX18
TBX18 MTE = 1.598
Null
rs76828289 - C - 6q16.1 - EPHA7
EPHA7 MTE = 1.722
Null
rs7557972 - T - 2q34 - PTH2R
PTH2R MTE = 1.602
Null
rs34460569 - T - 2q36.1 - PAX3
PAX3 MTE = 1.831
Null
rs9995821 - C - 4q31.3 - SFRP2
SFRP2 MTE = 1.644
Null
rs11462489 - A - 1p36.12 - EIF4G3
EIF4G3 MTE = 1.853
Null
rs4441951 - G - 6p21.1 - SUPT3H
SUPT3H MTE = 1.653
Null
rs72831074 - G - 10q25.3 - ABLIM1
ABLIM1 MTE = 1.655
Null
rs10773002 - A - 12q24.31 - CDK2AP1
CDK2AP1 MTE = 1.717
Null
Observed effects (χ^2)
Quantile of expected effects
t

Supplementary Figure 15. Facial traits associated map and multi–trait effects (MTE) of 57 face–associated SNPs.

57 C-GWAS identified study-wide suggestively significant regional lead SNPs are detailed with association pattern and projected effects distribution among all facial traits in order of ascending MTE. Each page contain 12 examples. The effect of the lead SNPs in terms of the *T* statistic from single-trait GWAS on 78 facial traits are superimposed on a face maps at left panel. Each map is named as 'lead SNP rsID - effect allele – loci position – the closest gene'. The squared projected effects (χ^2) are plotted against the quantile of the expected effects on the 26 projected vectors at right panel. The expected effects under the null are indicated using black dots connected by black lines. The observed effects of lead SNP are illustrated using orange diamonds connected by orange lines.

Supplementary Tables

Supplementary Table 1. Performance comparison between C–GWAS and mixAda, mixFisher, mixTippett and S_{Het}

For the four methods tested here for comparison which were not designed to run on parallel threads, we used R package “foreach” and “doParallel” as used in C-GWAS to parallel run them in 16 threads for acceleration.

	C–GWAS	mixAda	mixFisher	mixTippett	S_{Het}
Total time (16 threads, hour)	0.57	9.84	0.34	0.33	0.65
LD score intercept	0.997	1.041	1.04	1.045	1.073
Significant SNP number (<5e-8)	649	18	18	16	252
Identified significant loci (number)	16	12	9	8	16
Reported significant loci (number (%))	16 (100%)	1 (8%)	0 (0%)	1 (13%)	12 (75%)
Suggestive SNP number (<1.35e-6)	1043	160	137	107	880
Identified suggestive loci (number)	32	61	54	35	56
Reported suggestive loci (number (%))	22 (69%)	9 (15%)	8 (15%)	4 (11%)	18 (32%)

Supplementary Table 2. Computation time of C-GWAS modules in several scenarios

GWAS number	Thread number	Total time (s)	i-EbICoW time (s %)		TWT time (s %)		Other time (s %)	
5	1	4889	52	1.06%	4430	90.62%	407	8.33%
5	4	1918	18	0.96%	1640	85.53%	259	13.51%
5	16	1258	18	1.46%	1003	79.75%	236	18.78%
10	1	5501	151	2.74%	4699	85.43%	650	11.83%
10	4	2576	63	2.43%	2192	85.07%	322	12.49%
10	16	1451	52	3.55%	1133	78.03%	267	18.42%
20	1	9061	564	6.22%	6923	76.40%	1575	17.38%
20	4	3600	221	6.14%	2767	76.87%	611	16.98%
20	16	1709	166	9.72%	1218	71.23%	326	19.05%
30	1	12672	1437	11.34%	8754	69.09%	2481	19.57%
30	4	4280	449	10.49%	2983	69.69%	848	19.81%
30	16	2099	255	12.13%	1395	66.46%	449	21.41%
40	1	15840	2025	12.78%	9932	62.70%	3883	24.51%
40	4	5180	728	14.06%	3244	62.63%	1208	23.31%
40	16	2544	389	15.30%	1558	61.24%	597	23.46%
80	1	33509	8150	24.32%	13717	40.94%	11641	34.74%
80	4	10325	2522	24.42%	4460	43.20%	3343	32.38%
80	16	4554	1188	26.09%	1838	40.35%	1528	33.55%

Supplementary Table 3. The description of 13 facial landmarks

Landmarks	Abbreviation	Full name	Description
1	ExR	Right exocanthion	The point of right lateral outer canthus
2	EnR	Right endocanthion	The point of right lateral inner canthus
3	N	Nasion	The point where bridge of the nose meets forehead
4	Prn	Pronasale	The point of the nose tip
5	EnL	Left endocanthion	The point of left lateral inner canthus
6	ExL	Left exocanthion	The point of left lateral outer canthus
7	AlL	Left alare	The point of left lateral nose wing
8	Sn	Subnasale	The point where base of nasal septum meets philtrum
9	AlR	Right alare	The point of right lateral nose wing
10	Ls	Labial superius	The point of labial superius
11	ChR	Right cheilion	The point of right lateral angulus oris
12	Li	Labial inferius	The point of labial inferius
13	ChL	Left cheilion	The point of left lateral angulus oris
