# Supplementary material for: Combining genome-wide association studies highlight novel loci involved in human facial variation
Source: Nat Commun. 2022 Dec 20;13:7832. doi: 10.1038/s41467-022-35328-9 (PMC9767941; doi:10.1038/s41467-022-35328-9)
Supplement: Supplementary file 3 — Description of Additional Supplementary Files [file 41467_2022_35328_MOESM3_ESM.pdf]

Description of Additional Supplementary Files

File Name: Supplementary Data 1
Description: Supporting evidence of 13 novel loci identified by C-GWAS

File Name: Supplementary Data 2
Description: Study-wide suggestive significant results of C-GWAS and MinGWAS

File Name: Supplementary Data 3
Description: 56 C-GWAS suggestively significant regional lead SNPs and their replication results

File Name: Supplementary Data 4
Description: Study-wide suggestive significant results of C-GWAS and MTAG

File Name: Supplementary Data 5
Description: Significant results of C-GWAS and MinGWAS in gene ontology analysis

File Name: Supplementary Data 6
Description: Colocalizations between C-GWAS findings and eQTL in 22 tissues

File Name: Supplementary Data 7
Description: CNCC regulatory subnetwork based on C-GWAS and MinGWAS findings
