# Supplementary material for: Combining genome-wide association studies highlight novel loci involved in human facial variation
Source: Nat Commun. 2022 Dec 20;13:7832. doi: 10.1038/s41467-022-35328-9 (PMC9767941; doi:10.1038/s41467-022-35328-9)
Supplement: Supplementary file 5 — Reporting Summary [file 41467_2022_35328_MOESM5_ESM.pdf]

nature portfolio

Corresponding author(s): Fan Liu, Manfred Kayser

Last updated by author(s): Nov 13, 2022

Reporting Summary

Nature Portfolio wishes to improve the reproducibility of the work that we publish. This form provides structure for consistency and transparency in reporting. For further information on Nature Portfolio policies, see our Editorial Policies and the Editorial Policy Checklist.

Statistics

For all statistical analyses, confirm that the following items are present in the figure legend, table legend, main text, or Methods section.

n/a	Confirmed	
☐	☒	The exact sample size (*n*) for each experimental group/condition, given as a discrete number and unit of measurement
☐	☒	A statement on whether measurements were taken from distinct samples or whether the same sample was measured repeatedly
☐	☒	The statistical test(s) used AND whether they are one- or two-sided<br>*Only common tests should be described solely by name; describe more complex techniques in the Methods section.*
☐	☒	A description of all covariates tested
☐	☒	A description of any assumptions or corrections, such as tests of normality and adjustment for multiple comparisons
☐	☒	A full description of the statistical parameters including central tendency (e.g. means) or other basic estimates (e.g. regression coefficient) AND variation (e.g. standard deviation) or associated estimates of uncertainty (e.g. confidence intervals)
☐	☒	For null hypothesis testing, the test statistic (e.g. *F*, *t*, *r*) with confidence intervals, effect sizes, degrees of freedom and *P* value noted<br>*Give P values as exact values whenever suitable.*
☒	☐	For Bayesian analysis, information on the choice of priors and Markov chain Monte Carlo settings
☐	☒	For hierarchical and complex designs, identification of the appropriate level for tests and full reporting of outcomes
☐	☒	Estimates of effect sizes (e.g. Cohen's *d*, Pearson's *r*), indicating how they were calculated

Our web collection on statistics for biologists contains articles on many of the points above.

Software and code

Policy information about availability of computer code

Data collection	No software was used.
Data analysis	CGWAS is implemented as an open-source R package available at https://github.com/Fun-Gene/CGWAS. Other softwares used in data analysis includes: R package 'MPAT' (https://content.sph.harvard.edu/xlin/software.html#mpat, Liu et al., 2018); R package 'clusterProfiler' (Version 4.6.0, Yu et al., 2012); GREAT (http://great.stanford.edu/public/html/, Version 4, McLean et al., 2010); R package 'coloc' (Version 5.1.0, Giambartolomei et al., 2014); MTAG (https://github.com/omeed-maghzian/mtag, Turley et al., 2018); R package 'locuscomparer' (https://github.com/boxiangliu/locuscomparer, Liu et al., 2019); ISTAT (https://github.com/shahab-sarmashghi/ISTAT.git, Sarmashghi et al., 2019).

For manuscripts utilizing custom algorithms or software that are central to the research but not yet described in published literature, software must be made available to editors and reviewers. We strongly encourage code deposition in a community repository (e.g. GitHub). See the Nature Portfolio guidelines for submitting code & software for further information.

Data

Policy information about availability of data

All manuscripts must include a data availability statement. This statement should provide the following information, where applicable:

- Accession codes, unique identifiers, or web links for publicly available datasets
- A description of any restrictions on data availability
- For clinical datasets or third party data, please ensure that the statement adheres to our policy

C-GWAS p-values of the study-wide suggestively significant SNPs are provided in the Supplementary Data 2. Full C-GWAS summary statistics are publicly available via figshare at https://doi.org/10.6084/m9.figshare.21559086. Full GWAS summary statistics of the 78 facial traits used in discovery phase of C-GWAS application

are available on GWAS Catalog with the access number 31763980 at https://www.ebi.ac.uk/gwas/publications/31763980. The summary statistics of the Pennsylvania State University (PSU) and the Indiana University-Purdue University Indianapolis (IUPUI) are available on GWAS Catalog with the access number 33288918 at https://www.ebi.ac.uk/gwas/publications/33288918. The summary statistics of the National Survey of Physical Traits (NSPT), the Northern Han Chinese (NHC) and the Taizhou Longitudinal Study (TZL) are available on the National Omics Data Encyclopedia with NODE number OEP002283 at https://www.biosino.org/node/project/detail/OEP002283. The cis-eQTL results in 22 tissues were downloaded from GTEx V7 database at https://www.gtexportal.org/home/datasets. The regulatory network of CNCC is publicly available at https://github.com/AMSSwanglab/hReg-CNCC.

Field-specific reporting

Please select the one below that is the best fit for your research. If you are not sure, read the appropriate sections before making your selection.

☒ Life sciences ☐ Behavioural & social sciences ☐ Ecological, evolutionary & environmental sciences

For a reference copy of the document with all sections, see nature.com/documents/nr-reporting-summary-flat.pdf

Life sciences study design

All studies must disclose on these points even when the disclosure is negative.

Sample size	Sample size was determined by the amount of data available in published summary data and the cohort study. Details are provided in the Methods section of our manuscript
Data exclusions	Data exclusions were based on image quality and missing data, etc. Details are provided in Methods section of our manuscript.
Replication	Among the 17 novel loci identified by our C-GWAS, 13 are replicated by the combined evidence from six independently replication studies, the CNCC network analysis, or the colocalization analysis, as showed in Supplementary Data 1 and detailed in the Methods section.
Randomization	No randomization was performed. Since this is a population based study and did not focus on a treatment effect, randomization was not performed.
Blinding	No blinding was performed as this study is not experimental. Investigators did not have any access to identifying information.

Reporting for specific materials, systems and methods

We require information from authors about some types of materials, experimental systems and methods used in many studies. Here, indicate whether each material, system or method listed is relevant to your study. If you are not sure if a list item applies to your research, read the appropriate section before selecting a response.

Materials & experimental systems

n/a	Involved in the study	
☒	☐	Antibodies
☒	☐	Eukaryotic cell lines
☒	☐	Palaeontology and archaeology
☒	☐	Animals and other organisms
☐	☒	Human research participants
☒	☐	Clinical data
☒	☐	Dual use research of concern

Methods

n/a	Involved in the study	
☒	☐	ChIP-seq
☒	☐	Flow cytometry
☒	☐	MRI-based neuroimaging

Human research participants

Policy information about studies involving human research participants

Population characteristics	We included 1,174 Europeans of the Rotterdam Study for replication purpose, with a mean age of 74 years (SD=9). 52.6% of the sample was female. All other analysis was on summary data.
Recruitment	Details of the recruitment of the Rotterdam Study is provided in Methods section.
Ethics oversight	The Rotterdam Study has been approved by the Medical Ethics Committee of the Erasmus MC (registration number MEC 02.1015) and by the Dutch Ministry of Health, Welfare and Sport (Population Screening Act WBO, license number 1071272-159521-PG). The Rotterdam Study has been entered into the Netherlands National Trial Register (NTR; www.trialregister.nl) and into the WHO International Clinical Trials Registry Platform (ICTRP; www.who.int/ictrp/network/primary/en/) and under shared catalogue number NTR6831. All participants provided written informed consent to participate in the study and to have their information obtained from treating physicians.

Note that full information on the approval of the study protocol must also be provided in the manuscript.
